# Reduced myelin contributes to cognitive impairment in patients with monogenic small vessel disease

**DOI:** 10.1002/alz.70127

**Published:** 2025-05-02

**Authors:** Jannis Denecke, Anna Dewenter, Jongho Lee, Nicolai Franzmeier, Carolina Valentim, Anna Kopczak, Martin Dichgans, Lukas Pirpamer, Benno Gesierich, Marco Duering, Michael Ewers

**Affiliations:** ^1^ Institute for Stroke and Dementia Research (ISD) LMU University Hospital Munich Germany; ^2^ Laboratory for Imaging Science and Technology Department of Electrical and Computer Engineering Seoul Republic of Korea; ^3^ Department of Psychiatry and Neurochemistry Institute of Neuroscience and Physiology The Sahlgrenska Academy University of Gothenburg Gothenburg Sweden; ^4^ Munich Cluster for Systems Neurology (SyNergy) Munich Germany; ^5^ German Center for Neurodegenerative Disease (DZNE) Munich Germany; ^6^ Medical Image Analysis Center (MIAC) and Department of Biomedical Engineering University of Basel Basel Switzerland

**Keywords:** cerebral autosomal dominant arteriopathy with subcortical infarcts and leukoencephalopathy, chi separation, diffusion magnetic resonance imaging, susceptibility mapping, white matter hyperintensities

## Abstract

**INTRODUCTION:**

Myelin is pivotal for signal transfer and thus cognition. Cerebral small vessel disease (cSVD) is primarily associated with white matter (WM) lesions and diffusion changes; however, myelin alterations and related cognitive impairments in cSVD remain unclear.

**METHODS:**

We included 64 patients with familial cSVD (i.e., cerebral autosomal dominant arteriopathy with subcortical infarcts and leukoencephalopathy [CADASIL]) and 20 cognitively unimpaired individuals. χ separation applied to susceptibility weighted imaging was used to assess myelin and iron within WM hyperintensities, normal appearing WM, and two strategic fiber tracts. Diffusion‐based mean diffusivity and free water were analyzed for comparisons. Cognitive impairment was assessed by the Trail Making Test.

**RESULTS:**

CADASIL patients showed reduced myelin within WM hyperintensities and its penumbra in the normal appearing WM. Myelin was moderately correlated with diffusion and iron changes and associated with slower processing speed controlled for diffusion and iron alterations.

**DISCUSSION:**

Myelin constitutes WM alterations distinct from diffusion changes and substantially contributes to explaining cognitive impairment in cSVD.

**Highlights:**

- χ‐negative magnetic resonance signal was reduced within white matter hyperintensities and normal appearing white matter in patients with cerebral autosomal dominant arteriopathy with subcortical infarcts and leukoencephalopathy, suggesting widespread myelin decreases due to cerebral small vessel disease (cSVD).
- χ‐negative values were only moderately associated with diffusion tensor imaging derived indices including free water and mean diffusivity, suggesting that χ separation depicts distinct microstructural changes in cSVD.
- Alterations in χ‐negative values made a unique contribution to explain processing speed impairment, even when controlled for diffusion and iron changes.

## 1 BACKGROUND

Cerebral small vessel disease (cSVD) is thought to arise from a dysfunction in the perforating arterioles, capillaries, and venules of the brain.^1^ It is a major cause of ischemic stroke^2^, ^3^ and vascular dementia.^4^ White matter alterations are core disease‐defining brain abnormalities in cSVD, including among others macrostructural white matter hyperintensities (WMHs)^5^, ^6^ and microstructural changes in the normal appearing white matter (NAWM).^7^, ^8^ The underlying tissue components of white matter alterations in cSVD, however, remain unclear.^9^ Brain autopsy studies suggest that besides axonal degeneration, myelin loss is a major white matter alteration observed within areas of WMHs.^10^, ^11^, ^12^ Myelin is the lipid‐rich multi‐layer membrane enwrapping axons in the brain and crucial for signal transfer and axonal metabolic support.^13^, ^14^ In cSVD, endothelial dysfunction and reduced perfusion may contribute to myelin loss in the white matter.^15^, ^16^ Demyelination can occur without axonal degeneration,^17^ and has been associated with reduced working memory performance,^18^ suggesting that myelin damage could be an important contributor to cognitive decline. However, the extent of myelin loss throughout the brain in patients with cSVD has remained unclear so far. Magnetic resonance imaging (MRI) allows for brain‐wide assessment of myelin alterations, but requires advanced image acquisition protocols and post‐processing that limit accessibility.^19^, ^20^ The most commonly used MRI techniques for assessing microstructural white matter changes is diffusion tensor imaging (DTI). While DTI studies reported mean diffusivity (MD),^21^, ^22^ radial diffusivity (RD),^23^ and fractional anisotropy (FA) to be altered widespread in the brain of patients with cSVD,^24^, ^25^ DTI indices lack specificity to myelin.^20^, ^26^, ^27^, ^28^ The DTI findings were confirmed also when corrected for free water (FW),^29^ a model which assesses the presence of cerebrospinal fluid, for example, in edema,^30^ the reason why we include it in our analysis as well. Few studies have used advanced MRI indices to assess myelin in cSVD so far (for review see van den Brink et al.^31^). Specifically, some studies using myelin water fraction (MWF)^32^, ^33^ or magnetization transfer ratio imaging focused on white matter changes in normal aging^34^, ^35^, ^36^ or stroke^37^ and only few in diagnosed cSVD.^38^ So far, the most comprehensive study using multiparametric quantitative MRI indices (quantitative R1, R2*, and susceptibility maps) demonstrated myelin‐specific alterations in gray matter areas connected by WMH‐bearing tracts,^39^ leaving the question of white matter fiber tract demyelination in cSVD open. In summary, despite the pivotal importance of axonal myelin for cerebral signal transduction and thus cognitive function, there is a dearth of studies on assessing myelin alterations and their association with cognitive impairment in cSVD. Therefore, our major objectives were to (1) map myelin alterations both within WMHs and the NAWM in cSVD, (2), compare alterations in the measure of myelin alterations to those in standard DTI‐derived diffusion measures, and (3) test the association between myelin alterations and lower performance in processing speed, that is, a core cognitive deficit in cSVD. To assess myelin, we used susceptibility source separation called χ separation, that is, a recently developed technique applied to susceptibility weighted MR images.^40^, ^41^ This method uses the opposing magnetization of the two principal sources of susceptibility in the brain, myelin (diamagnetic) and iron (paramagnetic).^42^ The method separates the MRI signal of myelin from iron, which is crucial due to observed cerebral iron level changes in cSVD^43^, ^44^ and iron being a potential confounding factor for MR myelin measures.^45^, ^46^ We assessed χseparation along with DTI measures in patients with cerebral autosomal dominant arteriopathy with subcortical infarcts and leukoencephalopathy (CADASIL), a familial form of cSVD caused by a mutation in the *NOTCH3* gene.^47^ CADASIL recapitulates core MRI detectable white matter and cognitive abnormalities of sporadic cSVD.^48^, ^49^ Due to the early onset of white matter lesions typically occurring between the age of 20 and 40 years, CADASIL provides a unique opportunity to assess cSVD‐related brain changes at a time when older age–related and potentially confounding comorbidities are still seldom.^47^ To address our major aims, we (1) used χ separation to assess myelin within the WMH, NAWM, and a priori selected strategic fiber tracts underlying processing speed (i.e., forceps minor [FM] and left anterior thalamic radiation [ATR]);^50^ (2) compared the χ‐negative (myelin) alterations to those in standard DTI indices (MD, RD), FW, and χ‐positive (iron); and (3) tested the association between myelin alterations and processing speed while accounting for the effects of those other white matter indices.

## 2 METHODS

### 2.1 Participants

We included 64 CADASIL patients and 20 cognitively normal controls without CADASIL (CN) from the Vascular and Amyloid Predictors of Neurodegeneration and Cognitive Decline in Nondemented Subjects (VASCAMY)^22^ and Zooming in at Microvascular Malfunction in Small Vessel Disease Due to CADASIL with 7T MRI (ZOOM)^51^ studies conducted at the Institute for Stroke and Dementia Research (ISD), University Hospital Ludwig Maximilian University (LMU) Munich. Only participants with available T1‐weighted (T1w), fluid attenuated inversion recovery sequence (FLAIR), diffusion MRI, and T2^*^‐weighted multi‐echo gradient‐echo scans were considered. The diagnosis of CADASIL was confirmed by molecular genetic testing of the *NOTCH3* gene or via skin biopsy. Classification of the CN status was based on normal performance on the Consortium to Establish a Registry for Alzheimer's Disease (CERAD) test battery. Twenty CN participants matched by age, sex, and education to the CADASIL patients were selected from the VASCAMY and ZOOM studies, using the R package “MatchIt” (version 4.5.5).^52^ The matching was done to ensure that age was comparable between CADASIL and CN subjects as the CN subjects from the VASCAMY study tended to be older than the CADASIL patients (*n* = 30, mean age = 63.5, interquartile range [IQR] = 24, *t*[36.46] = –2.76, *p* = 0.009). After inclusion in the current study, two CADASIL patients had to be excluded because of problems warping their images to Montreal Neurological Institute (MNI) space and another two CADASIL patients because of alignment issues in the diffusion weighted imaging modality. Therefore, a total of 60 CADASIL patients were included in the statistical analyses.

### 2.2 Cognitive assessment

Processing speed was measured by the Trail Making Test (TMT) A and B completion time. The scores were stratified based on age and education.^53^ Compound scores for processing speed were first calculated as the mean of the stratified TMT A and B times and subsequently power transformed because of their inherent non‐normal distribution. We selected processing speed as the cognitive outcome in a hypothesis‐driven way, based on previous findings suggesting that predominantly processing speed is affected in cSVD.^50^ Nevertheless, we also report results for Mini‐Mental State Examination (MMSE) and CERAD in the supporting information.

**RESEARCH‐IN‐CONTEXT**

1. **Systematic review**: We systematically searched the PubMed library for articles on myelin alterations in cerebral small vessel disease (cSVD). Most neuroimaging studies focused on diffusion changes, which exhibits limited specificity to assess myelin. Therefore, myelin degeneration and its association with cognitive decline are not well understood in cSVD.
2. **Interpretation**: Our findings suggest extensive myelin degeneration in cSVD throughout the white matter, exceeding areas of gross white matter lesions. χ separation–assessed myelin alterations substantially contribute to explain key cognitive impairments, in addition to that by diffusion tensor imaging–derived markers.
3. **Future directions**: χ separation offers an attractive method to assess myelin that is applicable to standard susceptibility weighted imaging. Future studies may assess myelin alterations in sporadic SVD or neurodegenerative disease such as Alzheimer's disease in which SVD changes are highly prevalent. The generalizability in ethnically and socioeconomically more diverse samples remains to be tested.

### 2.3 MRI acquisition

Scans for both VASCAMY and ZOOM images were acquired on the same 3T Siemens Magnetom Skyra with 64‐channel head/neck coil (Siemens Healthineers).

For the two studies, comparable imaging protocols with only slight differences in the acquisition parameters were used. These included a 1 mm isotropic T1w scan, for VASCAMY: MP2RAGE (repetition time [TR] = 5500 ms, echo time [TE] 3.73 ms, flip angle = 7/4°, TI_1_ = 700 ms, TI_2_ = 2710 ms); ZOOM: MP‐RAGE (TR = 2500 ms, TE = 4.37 ms, flip angle = 7°, TI = 1100 ms). The same 1 mm isotropic 3D FLAIR (TR = 5000 ms, TE = 398 ms/393 ms, inversion time [TI] = 1800 ms) was used in both studies. Furthermore, 2 mm isotropic diffusion weighted images were acquired with an identical diffusion scheme of 10 b = 0 s/mm^2^, 30 b = 1000 s/mm^2^, and 60 b = 2000 s/mm^2^ directions. Both studies used a multi‐band acceleration (3x) and included the acquisition of one b = 0 image with inverted phase‐encoding direction to correct for susceptibility‐induced distortions. To perform the susceptibility source separation, a 3D T2*‐weighted multi‐echo gradient‐echo sequence with six roughly equidistant spaced echoes times and nearly identical parameters in both studies was used (VASCAMY: 0.8 × 0.8 × 2.0 mm, TR = 35 ms, TE = 4.92 ms, 9.85 ms, 14.70 ms, 19.60 ms, 24.57 ms, 29.50 ms, flip angle = 15°; ZOOM: 0.9 × 0.9 × 2.0 mm, TR = 35 ms, TE = 4.92 ms, 9.84 ms, 14.70 ms, 19.60 ms, 24.60 ms, 29.50 ms, flip angle = 15°). In both cases, the adaptive coil combine was run on the scanner and echoes were acquired in a bipolar readout mode.

### 2.4 Segmentation of WMH and lacunes

For all images the affine transform parameters to native T1w space were estimated using ANTS version 2.3.4.^54^ The T1w images were used to estimate translation parameters to MNI152 standard space and to a study‐specific white matter fiber orientation density distribution (WMFOD) template, to later extract tract‐based statistics (see section 2.8). Cortical white matter and cortical gray matter segmentations were acquired using SynthSeg, a pretrained contrast agnostic deep learning algorithm.^55^, ^56^ White matter masks were thresholded at a probability of 0.5 and eroded by 1 mm. Brain masks were extracted using HD‐BET version 1.0.0.^57^ Lacunes were segmented in T1w images using a seed‐growing algorithm after manual seed placement as described previously.^22^ WMH masks were segmented based on T2‐FLAIR images, using a histogram segmentation approach based on the Otsu method.^58^ The resulting WMH were manually edited to exclude artifacts. NAWM masks were generated by subtracting WMH masks from white matter masks. To assess the penumbra around WMHs we dilated the WMH mask in steps of two millimeters within NAWM areas, which resulted in successively larger NAWM rings around the WMHs, thus defining the distances within the penumbra. Lacunes were masked out from all maps. These masks of the different white matter areas were subsequently used as regions of interest (ROIs) to extract the measures of myelin, iron, and diffusion changes (see below).

### 2.5 χ separation to assess myelin and iron

We performed χ separation on T2^*^w multi‐echo phase and magnitude images to separate paramagnetic and diamagnetic sources using χ‐SepNet.^59^, ^60^ We first adjusted for the small shift caused by the bipolar readout by estimating the affine transformation parameters from the second to the first magnitude echo and applying them to all even magnitude and phase echoes (i.e. two, four, six). To avoid interpolation errors at the phase wraps, images were translated to their real and imaginary representation and upscaled to 1 mm isotropic resolution (the training resolution of the χ‐SepNet algorithm) using a sinc interpolation. Subsequently, the images were back‐transformed into magnitude and phase representations. The phase data were unwrapped using ROMEO^61^ and background field removal was performed using V‐SHARP from the STI suite.^62^ The R2^*^ signal was estimated from magnitude images using ARLO.^63^ Finally, the χ separation was performed using the abovementioned χ‐SepNet. A lower absolute χ‐negative score corresponds to a lower myelin level.

### 2.6 Computation of diffusion MRI metrics

Diffusion MRI scans were denoised^64^ and corrected for Gibbs ringing^65^ using MRtrix3 version 3.0.2.^66^ Next, the scans were corrected for susceptibility‐induced distortions via topup^67^ and for subject motion and eddy currents^68^, ^69^, ^70^ using eddy from the Functional Magnetic Resonance Imaging of the Brain (FMRIB) Software Library (FSL; version 6.0.3).^71^ To calculate the diffusion tensor model, the Diffusion Imaging in Python (DIPY) toolbox^72^ was used, and MD and RD were computed. To estimate FW, the bi‐tensor model of the DIPY implementation was used. It requires a multi‐shell acquisition scheme with multiple b0 images and is suitable for b‐values of up to 2000 s/mm^2^, after which the impact of non‐Gaussian diffusion becomes problematic.^30^, ^73^

### 2.7 Fiber‐tract segmentation

To determine the fiber tracts of interest including the FM and left ATR, we first constructed a WMFOD template, using a similar approach previously described.^74^ In short, we used multi‐shell multi‐tissue constrained spherical deconvolution to estimate the fiber orientation distribution (FOD). Deviating from the original approach, we registered all subjects to a group‐average template (using MRtrix3's population_template) and calculated an averaged FOD map. We used TractSeg^75^ to perform the white matter bundle segmentation and fiber tracking on the averaged FOD map, of which the FM and left ATR were of interest to this work. We a priori selected these two tracts because of our previous work showing lesions specifically in the FM and left ATR to be associated with processing speed decline in cSVD.^50^, ^76^

### 2.8 Extraction of ROI values

Next, we used MRtrix3 “tcksample” to extract χ‐negative, χ‐positive, MD, RD, and FW mean values along the streamlines of the FM and left ATR. In addition, we extracted these measures within the whole white matter mask, generated as described above. For the WMH ROIs, difference scores were computed such that for each CADASIL patient, the individual score in a WMH area was subtracted from the CN group average value in the spatially matched white matter area. We used the same procedure to obtain difference scores for each metric and WMH ROI including the WMH penumbra and the NAWM. For χ negative, a difference score < 0 means lower χ‐negative scores (reduced myelin) in CADASIL compared to the average score in the CN group. The computation of difference scores in spatially matched areas—rather than comparing lump sum score across different WMH locations—was necessary because white matter areas are typically myelinated in a region‐dependent manner in the normal brain, and thus group differences or ROI comparisons in myelin may be confounded by differences in the anatomical location of WMH.

### 2.9 Statistical analysis

Voxel‐based WMH and lacune frequency mapping was conducted by averaging the group‐specific binary lesion masks, such that the resulting map indicated the percentage of individuals with a specific lesion for a given voxel. We mapped group differences in χscores and DTI maps using voxel‐based analysis, applying an false discovery rate–adjusted significance threshold of *p* < 0.05 and a voxel extent threshold of 10 (SPM12 v7219).^77^ We compared χ‐negative values obtained from the whole white matter or tract‐ROIs between CADASIL and the control group by linear regression analyses, with group as the independent variable, controlled for age, sex, education, χ‐positive, and FW. Within the CADASIL group, the deviation of the subject‐level difference scores in χ‐negative from zero (indicating abnormal χ‐negative values) in the WMH, NAWM, and WMH penumbra ROIs were tested using one‐sample *t* tests. To test whether the χ‐negative differences were influenced by group differences in χ‐positive and FW, we adjusted the differences scores for age, sex, education, χ‐positive, and FW values. Briefly, we first estimated the regression coefficients of the covariates in a linear regression with χ‐negative scores as the dependent variable, and subsequently constructed the adjusted χ‐negative scores by adding the group‐averaged prediction to the individual residuals and then subtracting the subject‐specific CN average value according to the formula:

$$\;y_{diff}^{i}=\beta_{0}+\left(\sum_{j=1}^{p}\beta_{j}{\bar{y}}_{CADASIL}\right)+\varepsilon_{i}-{\bar{y}}_{CN}^{i},$$

where the number of CADASIL subjects ranges from *i = *1 to *n*; $\varepsilon_{i}$ refers to the subject‐level regression residual, ${\bar{y}}_{CN}^{i}$ is the mean CN‐group value for the *i*th subject's ROI definition, and $\beta_{j}$ are the regression coefficient for predictors ranging from *j *= 0 to *p*, including the intercept. We performed the same analysis steps for χ positive controlling for age, sex, education, and FW. The DTI indices were processed in the same fashion as well and adjusted for age, sex, education, and χ positive to dissociate iron from diffusion‐related alterations.

To assess the association between χ‐negative scores and DTI indices, we computed pairwise Pearson correlation coefficients within the CADASIL group and CN separately. To test the associations with processing speed, we used linear regression analyses including processing speed scores as the dependent variable and χ‐negative scores as the predictor variable along with age, sex, education, χ positive, MD, RD, FW, and intracranial volume–adjusted total WMH volume as covariates. Because of the collinearity between the MRI indices we used ridge regression analyses with an automatic estimate of the ridge parameter (R package “ridge,” version 3.3).^78^ For ridge regression models, *R* ^2^ was calculated as $R^{2}=1-\frac{SS_{E}}{SS_{T}}$ with $SS_{E}=\sum_{i=1}^{n}{\left(y_{i}-{\hat{y}}_{i}\right)}^{2}$ and $SS_{T}=\sum_{i=1}^{n}{\left(y_{i}-\bar{y}\right)}^{2}$. We report effect sizes as partial *R* ^2^ (p*R* ^2^) of the respective predictor calculated as $R_{partial}^{2}=\frac{t^{2}}{t^{2}+df}$ (R package “sensemakr,” version 0.1.4)^79^ or Cohen *d* in case of *t* tests. For ridge regression models, the residual degrees of freedom were estimated as effective $df=n-tr(2H-HH^{\prime }),$ with $H=X{\left(X^{\prime }X+\lambda I\right)}^{-1}X^{\prime }$ and $\hat{Y}=HY$.^80^ Furthermore, *p* values were calculated from a *t* distribution with the abovementioned residual degrees of freedom rather than the normal distribution as implemented in the “ridge” R package (which was intended for genetic studies with very large sample sizes in which a *t* distribution approximates a normal distribution, that is, a *t* distribution with infinite degrees of freedom). Results were considered significant below a nominal *p* value < 0.05. Visualizations were created with the R packages “ggplot2” (version 3.4.4)^81^ and “ggpubr” (version 0.6.0). ^82^

## 3 RESULTS

**FIGURE 1:**
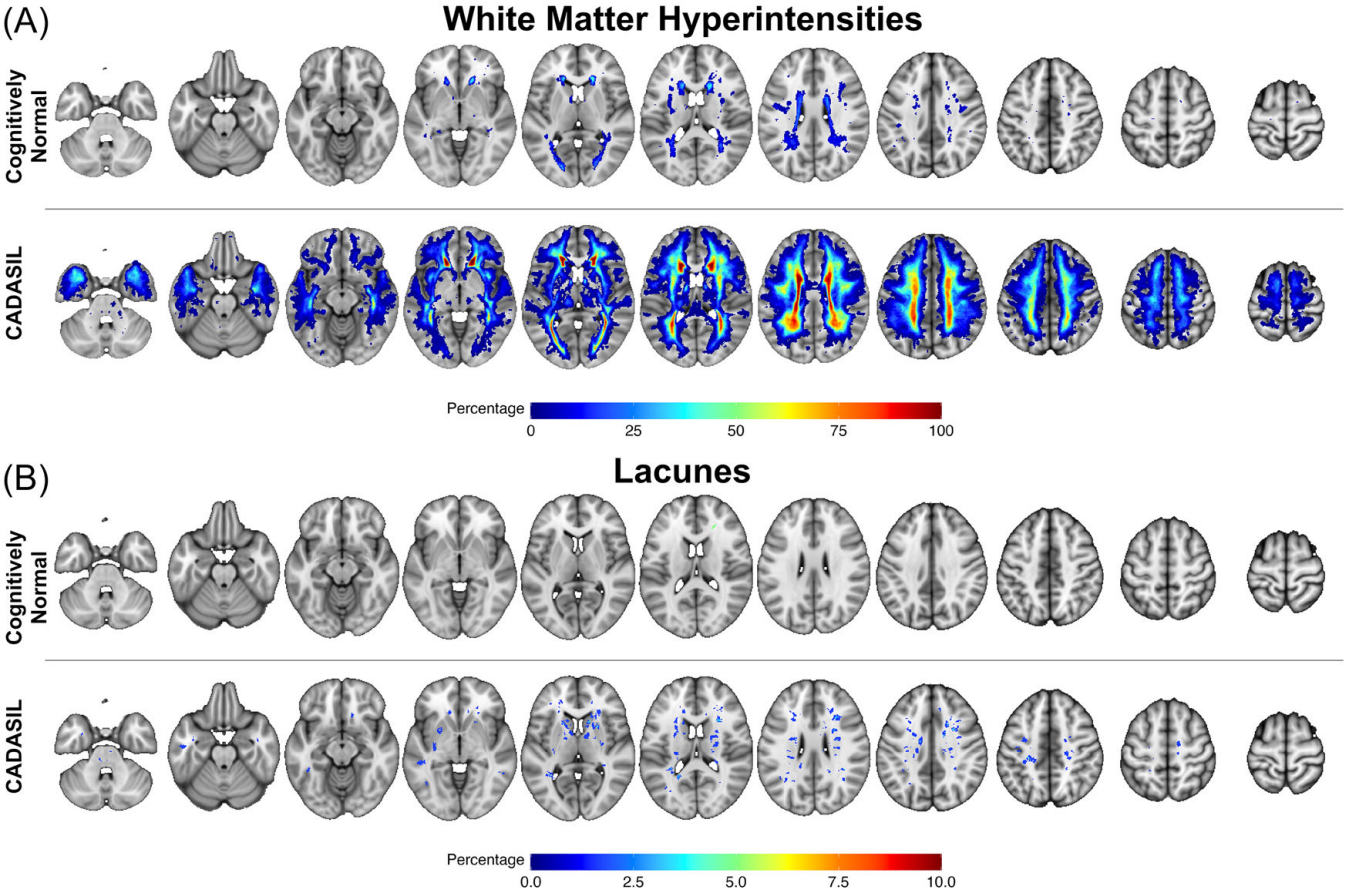
Spatial frequency of white matter hyperintensities and lacunar lesions. A, Frequency mapping of WMHs: the percentage of subjects with WMHs are mapped voxel‐wise onto axial slices for the CN group (upper row) and the CADASIL group (lower row). B, The frequency mapping for lacunes is shown in a corresponding way. Only voxels with a percentage > 0 are mapped. Left side is left hemisphere. CADASIL, cerebral autosomal dominant arteriopathy with subcortical infarcts and leukoencephalopathy; CN, cognitively normal; WM, white matter; WMH, white matter hyperintensity.

Demographics and descriptive statistics can be found in Table 1. The CADASIL group had significantly higher WMH volume (*t*[78] = 6.34, *p* < 0.001, *d* = 1.42) and a higher lacune count (*t*[78] = 3.57, *p* < 0.001, *d* = 0.80) compared to the CN group. Spatial frequency maps of the WMH and lacune within the CADASIL and CN are presented in Figure 1. Visual inspection showed that in the CADASIL group, WMHs were widely distributed within the white matter; CADASIL patients had WMHs at the anterior caps and > 90% at the rims around the lateral ventricles. In contrast, in the CN group, WMHs were spatially more limited with a maximum of 50% of CN individuals having a WMH at the left anterior cap. Lacunes were only present in CADASIL patients (except for a single lacune in one CN subject).

**TABLE 1:** Demographics and participant characteristics.

	Cognitively normal	CADASIL	Group difference
Group size	20	60	
Age, mean (IQR) (years)	58.4 (25)	54.23 (14)	*t*(78) = –1.34, *p* = 0.184, d = –0.30
Sex (%female)	40.0	61.7	χ^2^(1) = 0.80, *p* = 0.371
Education, mean (IQR) (years)	15.4 (5)	14.25 (3)	*t*(78) = –1.42, *p* = 0.159, d = –0.32
Processing speed, mean (IQR) (normalized)	0.08 (1.44)	−0.97 (1.03)	*t*(78) = –1.93, *p* = 0.057, d = –0.43
MMSE (IQR)	29.55 (1)	29.03 (1)	*t*(69) = –1.33, *p* = 0.187, d = –0.32
CERAD Memory, mean (IQR) (normalized)	1.67 (3.12)	−0.54 (4.17)	*t*(69) = –2.12, *p* = 0.038, d = –0.50
CERAD Figure Copy, mean (IQR) (normalized)	0.52 (0.49)	0.03 (1.43)	*t*(69) = –1.50, *p* = 0.138, d = –0.36
CERAD S‐Words, mean (IQR) (normalized)	1.17 (0.97)	−0.07 (1.27)	*t*(69) = –3.64, *p* < 0.001, d = –0.86
WMH volume, mean (IQR) (%, standardized by ICV)	0.19 (0.09)	5.98 (5.2)	*t*(78) = 6.34, *p* < 0.001, d = 1.42
Lacunes (count, IQR)	0.05 (0)	4.68 (7)	*t*(78) = 3.57, *p* < 0.001, d = 0.80
Lacunes, mean (IQR) (%, standardized by ICV)	0 (0)	0.0002 (0.0003)	*t*(78) = 3.19, *p* = 0.002, d = 0.71

*Note*. CERAD memory compound score is comprised of word list total, word list recall, word list recognition, and figure recall.

Abbreviations: CADASIL, cerebral autosomal dominant arteriopathy with subcortical infarcts and leukoencephalopathy; CERAD, Consortium to Establish a Registry for Alzheimer's Disease; ICV, intracranial volume; IQR, interquartile range, MMSE, Mini‐Mental State Examination; WMH, white matter hyperintensity.

### 3.1 χ‐negative index of myelin is reduced in WMH and NAWM in CADASIL

**FIGURE 2:**
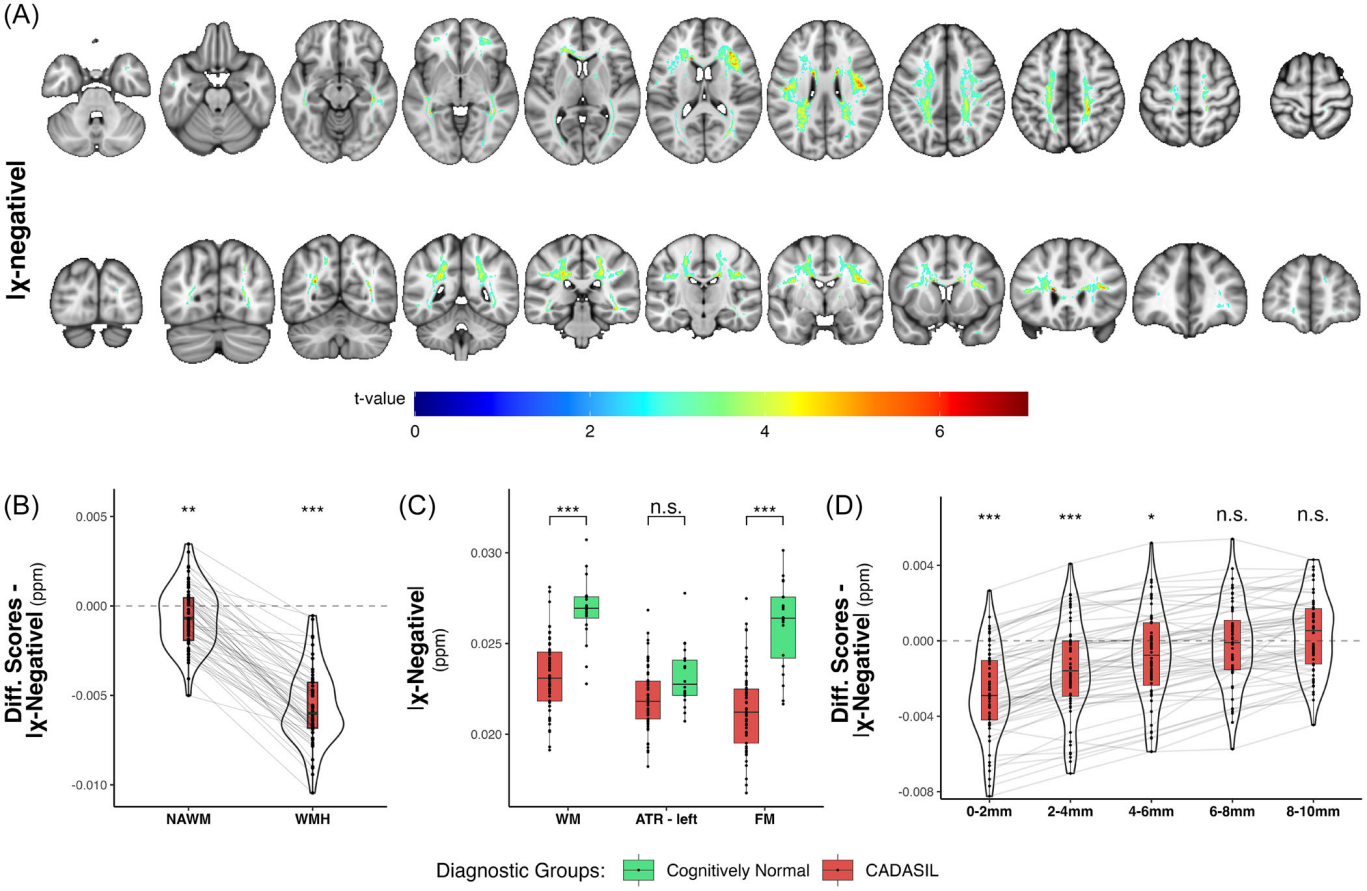
Voxel‐wise and ROI level descriptive results for χ negative showing group difference in CADASIL and CN controls. A, Significant voxel‐wise difference between CADASIL patients and CN of χ negative (CADASIL < CN) in the white matter mapped onto axial and coronal brain slices. Only voxels are plotted which survived false discovery rate correction on a level of α = 0.05 and a voxel extent threshold of 10. Testing for the opposite contrasts did not yield any significant cluster. Left side is left hemisphere. B, Violin plots with inserted box plots for χ‐negative difference scores extracted from NAWM and WMH areas. Each line represents the values for a CADASIL patient. C, Regular mean scores extracted from the global white matter, left ATR, and FM. D, Differences scores in χ‐negative values as a function of distance from WMH areas in the CADASIL group. Difference scores have been calculated in CADASIL from each subject's specific WMH/NAWM mask after they were corrected for age, sex, education, χ positive, and FW such that difference scores < 0 indicates lower χ‐negative scores (reduced myelin) in CADASIL compared to the average score in the CN group. Regular scores for the white matter and the two tracts are not given as differences scores, because these areas don't vary between individuals. For NAWM, WMH, and the WMH penumbra, one‐sample Welch *t* tests (*μ* = 0) were conducted and *p* values are plotted as asterisks (* *p* < 0.05, ** *p* < 0.01, *** *p* < 0.001). For the comparison of the white matter and tract scores, a regular linear model with a grouping factor was used, with age, sex, education, χ positive, and FW added as covariates. ATR, anterior thalamic radiation; CADASIL, cerebral autosomal dominant arteriopathy with subcortical infarcts and leukoencephalopathy; FM, forceps minor; NAWM, normal appearing white matter; ROI region of interest; WM, white matter; WMH, white matter hyperintensity.

Voxel‐based analysis showed a widely distributed decrease in χ‐negative scores in CADASIL, with peak group differences occurring within the FM and centrum semiovale (Figure 2A and Table S1 in supporting information). The CADASIL group showed significantly reduced χ‐negative scores in the whole white matter (*t*[73] = 3.5, *p *< 0.001, p*R* ^2^ = 0.15) and at the tract level in the FM (*t*[73] = 3.6, *p *< 0.001, p*R* ^2^ = 0.15) but not the left ATR (*t*[73] = 0.92, *p *= 0.358, p*R* ^2^ = 0.01) compared to the CN group. CADASIL patients showed abnormal χ‐negative difference scores in both NAWM and WMH areas (NAWM: *t*[59] = –2.67, *p* = 0.01, *d* = –0.34, WMH: *t*[59] = –20.54, *p* < 0.001, *d* = –2.65; see Figure 2B,C), suggesting that CADASIL exhibited a myelin decrease in both WMHs and NAWM.

### 3.2 χ‐negative alterations in the WMH penumbra

To test whether myelin alterations show an abrupt or gradual change when moving from the WMH area into the NAWM, we assessed the spatial gradient of χ‐negative alterations as a function of the distance from WMHs. We found that group differences in χ‐negative values varied along the spatial gradient, where a decrease in χ‐negative values in CADASIL patients was detectable until a distance of 6 mm from WMH (*t*[59] = –2.63, *p* = 0.011, *d* = –0.34; see Figure 2D and Table 2).

**TABLE 2:** Statistics for χ‐negative difference scores within the WMH penumbra.

WMH distance	*t*	*df*	*p*	*d*	95% CI
0–2 mm	−9.06	59	< 0.001***	−1.17	[−1.50, −0.84]
2–4 mm	−4.66	59	< 0.001***	−0.60	[−0.88, −0.32]
4–6 mm	−2.63	59	.011*	−0.34	[−0.60, −0.08]
6–8 mm	−0.68	59	.502	−0.09	[−0.34, 0.17]
8–10 mm	1.20	59	.235	0.15	[−0.10, 0.41]

*Note*. Statistical results from one‐sample Welch *t* tests (*μ* = 0) for χ negative in the penumbra of WMH. Subject‐specific scores were corrected for age, sex, education, χ positive, and FW before the calculation of the difference scores. Only NAWM areas without lacunar lesions were assessed.

Abbreviations: CI, confidence interval; NAWM, normal appearing white matter; WMH, white matter hyperintensity.

* *p* < 0.05.

** *p* < 0.01.

*** *p* < 0.001.

### 3.3 DTI metrics are altered but weakly associated with χ negative

We performed corresponding analyses for DTI‐based MD, RD, and FW. The full details can be found in Figures S1 through S3 and Table S2 in supporting information. Auxiliary results for FA are presented in the supporting information for completeness, but not further discussed here (see Figure S4 in supporting information). For all diffusion indices we found significant increases in CADASIL in all assessed ROIs including the global white matter, WMH, NAWM, and both fiber tracts (all *p* values < 0.001). For the spatial gradient analysis, increased MD and FW values were present up to the maximally assessed distance of 10 mm in the WMH penumbra, and up to 8 mm for RD. For χ‐positive values, the results were more mixed with significantly reduced χ‐positive values in the NAWM, WMH, the left ATR, and the penumbra up to 10 mm (all *p* values < 0.05; see Figure S5 in supporting information), but not the global white matter and the FM.

**FIGURE 3:**
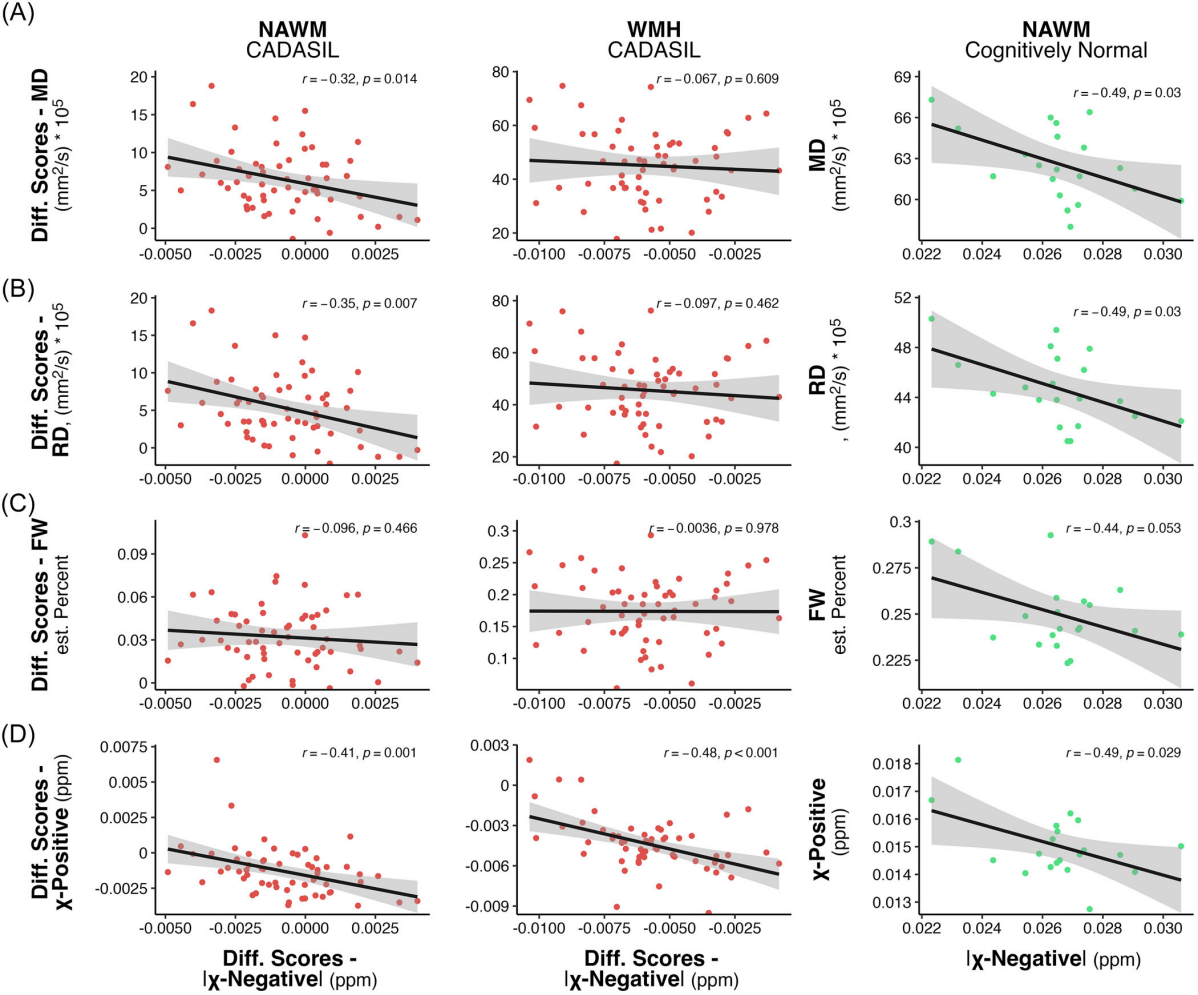
Association between χ negative and each MD, RD, FW, and χ positive in NAWM and WMH. Scatterplot for the association between χ negative and MD (A), RD (B), FW (C), χ positive (D) for the CADASIL group in NAWM (left column) and WMH (middle column) and the CN group in NAWM (right column). Regression line and 95% CI (shaded area) are shown. ATR, anterior thalamic radiation; CADASIL, cerebral autosomal dominant arteriopathy with subcortical infarcts and leukoencephalopathy; CI, confidence interval; FM, forceps minor; FW, free water; MD, mean diffusivity; NAWM, normal appearing white matter; RD, radial diffusivity; ROI region of interest; WM, white matter; WMH, white matter hyperintensity.

Next, to test whether the observed alterations in χ‐negative and DTI indices are linked, we estimated the shared variance of χ‐negative and DTI indices in the NAWM and WMH areas. For the CADASIL group, lower χ‐negative values were moderately associated with higher MD values in the NAWM (*r*[58] = –0.32, *p* = 0.014), and did not reach significance in the WMH areas (*r*[58] = –0.07, *p* = 0.61, Figure 3A), suggesting at best a weak association between both measures in CADASIL. Similarly, higher RD were associated with lower χ negative in the NAWM (*r*[58] = –0.35, *p *= 0.007) but not in the WMH areas (*r*[58] = –0.10, *p *= 0.46, Figure 3B). In contrast, FW was not significantly associated with χ negative in CADASIL (NAWM: *r*[58] = –0.097, *p* = 0.46, WMH: *r*[58] = –0.004, *p* = 0.98, Figure 3C).

For the CN group, we found a significant association between χ‐negative and MD (*r*[18] = –0.49, *p *= 0.03) or RD values (*r*[18] = –0.49, *p *= 0.03, Figure 3, last column), suggesting that ≈ 25% of the variance in χ‐negative values could be explained by variances in MD or RD values. The association with FW was not significant despite its moderate effect size (*r*[18] = –0.44, *p *= 0.053). Because CN exhibited only few WMHs, the association was not assessed in those areas. χ‐positive values were inversely associated with χ‐negative values in WMH and NAWM for both groups (*r* between –0.41 and –0.49, all *p* values < 0.05, Figure 3D).

### 3.4 χ negative is associated with processing speed independently from diffusion alterations

**FIGURE 4:**
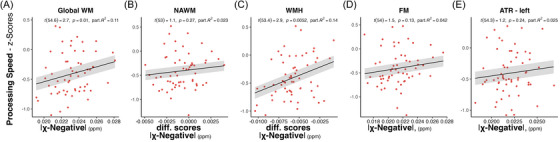
Association between χ‐negative scores in white matter regions and processing speed. Residual regression plots for different ROIs (A–E) from ridge regression models for processing speed scores as a function of χ‐negative scores in patients with CADASIL. Regression line and 95% confidence level (shaded area) are shown. ATR, anterior thalamic radiation; CADASIL, cerebral autosomal dominant arteriopathy with subcortical infarcts and leukoencephalopathy; FM, forceps minor; NAWM, normal appearing white matter; ROI region of interest; WM, white matter; WMH, white matter hyperintensity.

We separately assessed the predictive values of χ negative in the global white matter, NAWM, WMH, and the two tracts on processing speed in the CADASIL patients. Results are shown in Table 3 and Figure 4. Lower χ‐negative values in the global white matter (χ negative: *t*[54.6] = 2.65, *p* = 0.010, p*R* ^2^ = 0.11) and in WMH (χ negative: *t*[53.4] = 2.91, *p* = 0.005, p*R* ^2^ = 0.14) were associated with lower processing speed, controlled for age, sex, education, MD, RD, FW, χ‐positive, and intracranial volume–adjusted total WMH volume. χ‐negative values were neither at the tract level nor in NAWM regions associated with processing speed. For sensitivity and specificity comparisons we also provide results for the MMSE and CERAD subscales. We find a significant association for CERAD memory compound score and phonemic fluency (S words) with χ negative in WMH (see Figure S6 and Tables S3–S6 in supporting information).

**TABLE 3:** Statistics for predicting processing speed split by WM ROIs.

WM region/predictors	Est. (scaled)	SE (scaled)	*t* value	*df*	*p* value	Partial *R* ^2^
**Global WM**						
|χ‐negative|	0.63	0.24	2.65	54.6	0.010*	0.11
MD	−0.39	0.11	3.53	54.6	0.001***	0.19
RD	−0.40	0.11	3.49	54.6	0.001***	0.18
FW	−0.08	0.15	0.52	54.6	0.604	0.00
χ‐positive	−0.15	0.24	0.65	54.6	0.521	0.01
WMH volume	0.28	0.18	1.53	54.6	0.131	0.04
Age (years)	−0.18	0.24	0.73	54.6	0.466	0.01
Sex (male)	−0.15	0.24	0.60	54.6	0.548	0.01
Education (years)	0.62	0.24	2.56	54.6	0.013*	0.11
**NAWM**						
|χ‐negative| Diff. Score	0.31	0.28	1.12	53	0.267	0.02
RD diff. score	−0.80	0.23	3.51	53	0.001***	0.19
MD diff. score	−0.65	0.17	3.87	53	< 0.001***	0.22
FW diff. score	0.14	0.26	0.55	53	0.586	0.01
χ‐positive diff. score	0.06	0.28	0.21	53	0.835	0.00
WMH volume	0.17	0.28	0.62	53	0.535	0.01
Age (years)	−0.19	0.28	0.69	53	0.493	0.01
Sex (male)	−0.32	0.28	1.15	53	0.255	0.02
Education (years)	0.64	0.28	2.28	53	0.026*	0.09
**WMH**						
|χ‐negative| diff. score	0.95	0.33	2.91	53.4	0.005**	0.14
RD diff. score	−0.48	0.19	2.52	53.4	0.015*	0.11
MD diff. score	−0.41	0.18	2.27	53.4	0.028*	0.09
FW diff. score	0.42	0.26	1.61	53.4	0.113	0.05
χ‐positive diff. score	−0.34	0.34	0.99	53.4	0.327	0.02
WMH volume	−0.34	0.35	0.97	53.4	0.339	0.02
Age (years)	−0.20	0.33	0.60	53.4	0.549	0.01
Sex (male)	−0.19	0.32	0.58	53.4	0.564	0.01
Education (years)	0.92	0.32	2.91	53.4	0.005**	0.14
**FM**						
|χ‐negative|	0.42	0.28	1.54	54	0.130	0.04
RD	−0.46	0.15	3.17	54	0.002**	0.16
MD	−0.51	0.16	3.24	54	0.002**	0.16
FW	0.04	0.22	0.20	54	0.845	0.00
χ‐positive	0.05	0.27	0.19	54	0.852	0.00
WMH volume	0.22	0.26	0.85	54	0.400	0.01
Age (years)	−0.17	0.28	0.62	54	0.540	0.01
Sex (male)	−0.26	0.28	0.95	54	0.346	0.02
Education (years)	0.63	0.28	2.26	54	0.028*	0.09
**ATR‐left**						
|χ‐negative|	0.28	0.24	1.18	54.3	0.241	0.03
RD	−0.48	0.13	3.83	54.3	< 0.001***	0.21
MD	−0.47	0.12	3.88	54.3	< 0.001***	0.22
FW	−0.38	0.16	2.34	54.3	0.023*	0.09
χ‐positive	−0.47	0.24	1.98	54.3	0.053	0.07
WMH volume	0.32	0.22	1.43	54.3	0.159	0.04
Age (years)	−0.16	0.24	0.70	54.3	0.490	0.01
Sex (male)	−0.10	0.24	0.40	54.3	0.692	0.00
Education (years)	0.60	0.24	2.51	54.3	0.015*	0.10

*Note*: Statistical results for stratified and power transformed processing speed scores (TMT A+B). Negative values indicate worse test performance. WMH and NAWM models represents difference scores from within individual WMH and NAWM areas. Global WM, FM, and left ATR represents scores from the respective tracts, regardless of WMH or NAWM. WMH volume was standardized by the total intracranial volume.

Abbreviations: ATR, anterior thalamic radiation; FM, forceps minor; FW, free water; MD, mean diffusivity; NAWM, normal appearing white matter; RD, radial diffusivity; ROI, region of interest; SE, standard error; TMT, Trail Making Test; WM, white matter; WMH, white matter hyperintensity.

* *p* < 0.05.

** *p* < 0.01.

*** *p* < 0.001.

## 4 DISCUSSION

The major findings of the current study in CADASIL patients were (1) χ‐negative values were decreased in WMHs and NAWM, where the decrease followed a spatial gradient relative to the WMH distance; (2) alterations in χ negative were distinct from those in iron (χ‐positive) and diffusion (MD, FW); and (3) the decrease in χ negative contributed to explain slower cognitive processing beyond effects of MD, FW, and χ positive. These results suggest that genetically caused cSVD is associated with widespread myelin alterations not explainable by changes in iron and diffusion. They substantially contribute to the decrease in mental processing beyond diffusion alterations.

Regarding our first major finding, voxel‐based mapping and ROI‐based approaches indicated a heterogenous distribution of myelin decreases in cSVD. The strongest reductions were located within areas of WMHs, consistent with previous histochemical findings.^9^, ^10^, ^11^ The mapping of the χ‐negative alterations showed a pronounced decrease in the FM and in the centrum semiovale. Those overlap with the vascular territories of the anterior and middle cerebral arteries, both previously associated with increased vulnerability to the occurrence of WMHs.^83^ Therefore, these areas may be particularly susceptible to cSVD‐related hypoperfusion,^84^ and thus the development of WMHs and associated demyelination.^85^, ^86^, ^87^

The observed χ‐negative alterations extended beyond WMH borders, with reduction size following a spatial gradient decreasing with distance from WMH borders. Similarly, diffusion alterations were strongest near WMHs, levelling off within the penumbra, as also described by others.^88^, ^89^, ^90^ However, divergent results for myelin were recently reported, suggesting that selectively diffusion but not myelin is changed within the penumbra of WMHs.^91^ This discrepancies could be due to different patient populations (stroke survivors vs. CADASIL) or differences in the measures of myelin (myelin water fraction imaging vs. χ separation). Furthermore, penumbra effects likely depend on the spatial definition and technical limitations of detecting WMHs^92^ and differences in their segmentation^93^ may have contributed to these discrepancies. Overall, regarding current and previous findings it is fair to say that myelin alterations in cSVD extend beyond WMHs, where the variability in the penumbra remains to be clarified.

For our second major finding comparing changes in χ negative with iron and diffusion indices, we observed expected changes in all of them, consistent with previous studies on diffusion and iron^12^, ^94^, ^95^ in CADASIL and cSVD (for review see van den Brink et al.^31^). In particular, MD was highly sensitive to microstructural white matter changes^22^ and correlated with cognitive decline.^96^ However, increases in MD were strongly driven by FW.^29^, ^97^ These could stem from extracellular pools of unbound water in edema^29^ and intramyelin vacuolation occurring in cSVD including CADASIL.^10^, ^98^ Therefore, MD may be sensitive for microstructural white matter changes, but not specifically for myelin. We also found increased RD, which measures diffusion perpendicular to the tract. Previous studies suggest RD as a potential marker of myelin.^99^, ^100^ However, FW correction resulted in both regional increases and decreases of RD in aging and disease,^101^, ^102^ making the interpretation of RD in terms of myelin changes difficult.^28^, ^103^ This is also suggested by a recent meta‐analysis, in which myelin water fraction and magnetization transfer ratio correlated better with *post mortem* myelin staining, compared to RD.^104^ In our study, χ negative was not associated with RD, suggesting that they reflect different tissue changes. We controlled our analysis for FW, demonstrating that χ negative is consistently decreased in CADASIL. Overall, our results suggest that χ‐negative values cannot be explained by diffusion changes but rather reflect a distinct tissue component of white matter alterations. While diffusion markers may be more sensitive regarding white matter alterations, they are not specific to myelin damage. χ separation is based on a biophysical model that attributes magnetization to known physical properties of myelin versus iron, thus rendering high construct validity.^40^ χ separation detected myelin in ex vivo MRI spatially corresponds to histochemical ex vivo myelin stainings,^40^ and demonstrated sensitivity toward white matter lesion in primary demyelinating diseases such as multiple sclerosis,^105^, ^106^ which was also confirmed in an ex vivo study.^107^ Therefore, our findings of χ‐negative alterations independent of iron or diffusion changes suggest demyelination in cSVD and support the utility of χ separation in assessing myelin. Future brain autopsy studies, however, need to verify whether χ‐negative changes reflect microstructural white matter alterations limited to myelin in contrast to diffusion changes that may reflect broader tissue alterations in the WM.

For our third major finding, we demonstrated that reduced χ‐negative values in the whole white matter and WMH are associated with lower processing speed in CADASIL patients. Our findings align with previous studies linking lower MWF to reduced cognitive performance in aging and stroke.^38^, ^108^ We significantly advance these findings, demonstrating that this association cannot be explained by diffusion or potential confounding factors like FW or iron (χ positive) in cSVD. At the tract level, diffusion indices in the left ATR and FM were linked to lower processing speed, aligning with previous findings associating white matter lesions in these tracts to processing speed in cSVD.^76^ While χ negative was also decreased in the FM, this was not associated with processing speed. Rather, myelin alterations in the global white matter and in particular WMH were associated with processing speed, suggesting a wider network of white matter tracts to be involved in processing speed changes in cSVD.^109^ Myelin is crucial for fine tuning temporal synchronicity of signal transduction within functional brain networks.^110^ Genetically or drug‐induced myelin damage in mice increased resting state functional MRI‐assessed functional disruptions, associated with behavioral impairments in a functional network–dependent manner.^111^ Therefore, cSVD‐related demyelination potentially disrupt functional networks, leading to reduce mental processing speed. The current findings encourage future studies to address demyelination‐related functional network dysconnectivity in cSVD.

Myelin alterations are widespread in cSVD, contributing to processing speed impairments, a core deficit of executive function that cannot be explained only by diffusion or iron changes. A practical advantage is that χ separation can be applied to standard multi‐echo gradient echo images, widely available on different MRI scanner brands, and does not require advanced MRI physics expertise for its implementation in contrast to other myelin‐sensitive MR including MWF^32^, ^33^, ^112^, ^113^ (for excellent reviews on alternate MRI‐based myelin measures see van der Weijden et al.,^19^ Lee et al.,^20^ and Piredda et al.^114^). Therefore, χ separation may be an attractive tool for a wider implementation to assess myelin changes in cSVD and Alzheimer's disease, in which cSVD and associated demyelination frequently occur.^115^, ^116^

## 5 LIMITATIONS

Some potential caveats should be considered when interpreting our findings. First, χ separation is promising for assessing myelin but has potential confounding influences,^41^ for example the fiber tract orientation.^117^ This effect may be strongest when comparing myelin levels between brain regions. We held white matter location constant by computing difference scores in spatially matched locations to reduce spatial variation influence. Furthermore, image reslicing potentially results in penumbra‐like effects around WMHs, though the impact would be largest at 0 to 2 mm. We still observed significant alteration at 4 to 6 mm, where reslicing effects are unlikely to account for the myelin alterations. Furthermore, in auxiliary analysis we calculate the penumbra scores from subject native space and corresponding MNI space HC maps, avoiding warping of the CADASIL patient images. Albeit resulting in small numerical differences, the penumbra effects remained (Figure S7 and Table S7 in supporting information). Second, although CADASIL recapitulates many sporadic cSVD features,^118^ differences exist, like increased WMH in the temporal lobe of CADASIL patients^119^ with underlying tissue composition potentially varying in atypical areas.^120^ However, we observed myelin alterations also in shared WMH areas such as the frontal lobe. Third, the DTI model was fit including the b = 2000 s/mm^2^ shell at which signal distribution is not strictly Gaussian, likely causing a slight underestimation of the diffusion signal.^121^, ^122^ However, these were the lowest two b‐value shells available, required for the multi‐shell FW model. Fourth, we acknowledge that our number of CN would ideally be higher; however, an age‐matched group of CN was prioritized. Overall, it remains to be seen whether the current findings can be generalized to sporadic cSVD. Last, we caution that the current cohort exhibits limited social and ethnic diversity. The endophenotype of cSVD may potentially vary by ethnic background;^123^ for example, CADASIL patients with Asian ethnicity showed a somewhat higher occurrence of lacunes compared to White patients.^124^ Given the rare population frequency of CADASIL, the assessment of socially and ethnically more diverse patients was beyond the scope of this study.

## 6 CONCLUSIONS

We showed that χ separation in the whole white matter or WMH explained up to 14% of processing speed decline, suggesting that myelin changes are important for understanding cognitive decline in cSVD. Our findings encourage future studies to investigate myelin as a potential target to alleviate cognitive symptoms. Myelination shows plasticity even in adulthood, when lifestyle activities in adults^125^ and higher physical activity in either older individuals with cSVD^126^ or patients with a history of stroke^127^ were associated with higher myelin levels. However, whether exercise enhances myelin levels and thus alleviates cognitive deficits in cSVD remains to be shown. Demyelination can be targeted by drugs already approved for other diseases and which may stimulate oligodendrocyte precursor cells and remyelination.^128^, ^129^ Therefore, repurposing such drugs for cSVD is a potential strategy, for which χ separation may detect and track myelin alterations. However, future longitudinal studies are needed to investigate myelin alterations rates in both CADASIL and sporadic cSVD.

## CONFLICT OF INTEREST STATEMENT

Jannis Denecke: received travel funding directly to LMU for presenting parts of this work at an international scientific conference by the Alzheimer Forschung Initiative e.V. Anna Dewenter: no funding was received toward this work. Jongho Lee: Declares a patent on the χ‐separation method. Nicolai Franzmeier: received consulting honoraria from MSD as well as speaker honoraria from Life Molecular Imaging, GE Healthcare, and Esai. Carolina Valentim: no funding was received toward this work. Anna Kopczak: no funding was received toward this work. Martin Dichgans: no funding was received toward this work. Lukas Pirpamer: no funding was received toward this work. Benno Gesierich: no funding was received toward this work. Marco Duering: served on a scientific advisory board for Biogen, an adjudication board for Hovid Berhad, and as a consultant for Roche and received speaker honoraria from Sanofi Genzyme. Michael Ewers: received research support from Eli Lilly.

## CONSENT STATEMENT

Both studies, ZOOM and VASCAMY, were approved by the local ethics committee of the LMU, and written informed consent was acquired from all participants.

## Supporting information



Supporting information

Supplementary material
Supplementary material is available at* Alzheimer's & Dementia* online.

## Data Availability

The data for the VASCAMY study is available upon request from the corresponding author, but not publicly due to privacy restrictions. The ZOOM data can be requested from the cSVDs@Target group.
