# Supplementary material for: Reduced myelin contributes to cognitive impairment in patients with monogenic small vessel disease
Source: Alzheimers Dement. 2025 May 2;21(5):e70127. doi: 10.1002/alz.70127 (PMC12046978; doi:10.1002/alz.70127)
Supplement: Supplementary file 2 — Supplementary material Supplementary material is available at Alzheimer's & Dementia online. [file ALZ-21-e70127-s001.pdf]

Supplementary information

Detailed Results for DTI Indices

Voxel-based group comparisons showed peaks of abnormally elevated MD located in the FM and at the border of both lateral ventricles in the centrum semiovale (Supplementary Fig. E1 A), where the white matter areas of significant MD increases encompassed those of χ-negative values. On an ROI level, CADASIL patients also showed abnormal values in all ROIs tested (white matter: $t(74) = -8.0$, $p < .001$, $pR^2 = .47$, FM: $t(74) = -8.3$, $p < .001$, $pR^2 = .48$, ATR-left: $t(74) = -7.8$, $p < .001$, $pR^2 = .45$, NAWM: $t(59) = 12.66$, $p < .001$, $d = 1.63$, WMH: $t(59) = 26.83$, $p < .001$, $d = 3.46$), see supplementary Fig. E1 B&C. For the penumbra around WMH, MD scores significantly differed for the maximally assessed range of 10 mm ($t(59) = 4.80$, $p < .001$, d = 0.62, see supplementary Fig. E1 D and supplementary Table E2). All the tests for MD were controlled for age, sex, education, and χ-positive in the same manner as the main analysis for χ-negative.

The findings for FW mirrored the findings for MD both in shape (Supplementary Fig. E2 A) and in significance (white matter: $t(74) = -8.7$, $p < .001$, $pR^2 = .51$, FM: $t(74) = -9.1$, $p < .001$, $pR^2 = .53$, ATR-left: $t(74) = -8.3$, $p < .001$, $pR^2 = .48$, NAWM: $t(59) = 11.36$, $p < .001$, $d = 1.47$, WMH: $t(59) = 25.68$, $p < .001$, $d = 3.31$), see supplementary Fig. E2 B&C. The penumbra also still differed at 10 mm ($t(59) = 5.22$, $p < .001$, d = 0.67, see supplementary Fig. E2 D and supplementary Table E2). Again, all analyses were controlled for age, sex, education, and χ-positive.

For RD the results were also very similar to MD and FW (Supplementary Fig. E3 A, white matter: $t(74) = -8.1$, $p < .001$, $pR^2 = .47$, FM: $t(74) = -8.7$, $p < .001$, $pR^2 = .50$, ATR-left: $t(74) = -7.7$, $p < .001$, $pR^2 = .45$, NAWM: $t(59) = 9.96$, $p < .001$, $d = 1.29$, WMH: $t(59) = 26.56$, $p < .001$, $d = 3.43$), see supplementary Fig. E3 B&C. One difference however was that the extent of the penumbra effect was slightly reduced with significant differences only up until 8 mm ($t(59) = 5.75$, $p < .001$, d = 0.74, see supplementary Fig. E3 D and supplementary Table E2). Again, all analyses were controlled for controlled for age, sex, education, and χ-positive.

Detailed results for χ-positive

For χ-positive scores, we found significantly lower scores for CADASIL patients mostly in the centrum semiovale (Supplementary Fig. E2 A), however to a much smaller extent than for χ-

negative and the DTI indices. Furthermore, there was barely any overlap between the significantly changed areas of χ-negative and χ-positive. On the ROI level the results also slightly differed, but χ-positive was still significantly lower in the NAWM, WMH and the left ATR. (white matter: $t(74) = 0.98$, $p = .33$, $pR^2 = .01$, FM: $t(74) = 0.88$, $p = .38$, $pR^2 = .01$, ATR-left: $t(74) = 2.3$, $p = .022$, $pR^2 = .07$, NAWM: $t(59) = -8.07$, $p < .001$, $d = -1.04$, WMH: $t(59) = -17.49$, $p < .001$, $d = -2.26$), see supplementary Fig. E4 B&C. In the WMH penumbra, χ-positive scores were significantly lower at all assessed distances, however by visual inspection, it did not seem like they gradually regress back to normal values (see supplementary Fig. E4 D and supplementary Table E2). All the tests were adjusted for age, sex, education, and FW.

Table E1 Cluster peaks for group differences in χ-negative.

Sig. Cluster / Peak	x	y	z	Tract	Hemisphere	*p*-value
Cluster 1, peak 1	-14	26	12	Forceps minor	left	< 0.001
Cluster 1, peak 2	-14	18	19	Forceps minor	left	< 0.001
Cluster 1, peak 3	-12	29	4	Forceps minor	left	0.001
Cluster 2, peak 1	38	-39	-7	Inferior Fronto-Occipital Fasciculus	right	0.001
Cluster 2, peak 2	42	-9	27	Superior Longitudinal Fasciculus	right	0.001
Cluster 2, peak 3	15	-5	29	Forceps minor	right	0.001

Note. Cluster peaks for group differences in χ-negative. SPM12 suggests three peaks per cluster. Therefore, not all local peaks may be indicated. Coordinates are given for the MNI ICBM 152 template. Tracts are based on the JHU ICBM atlas. Where no atlas value was given, the closest tract was used.

Figure E1 Voxel-wise and ROI level descriptive results for mean diffusivity showing group difference in CADASIL and cognitively normal controls.

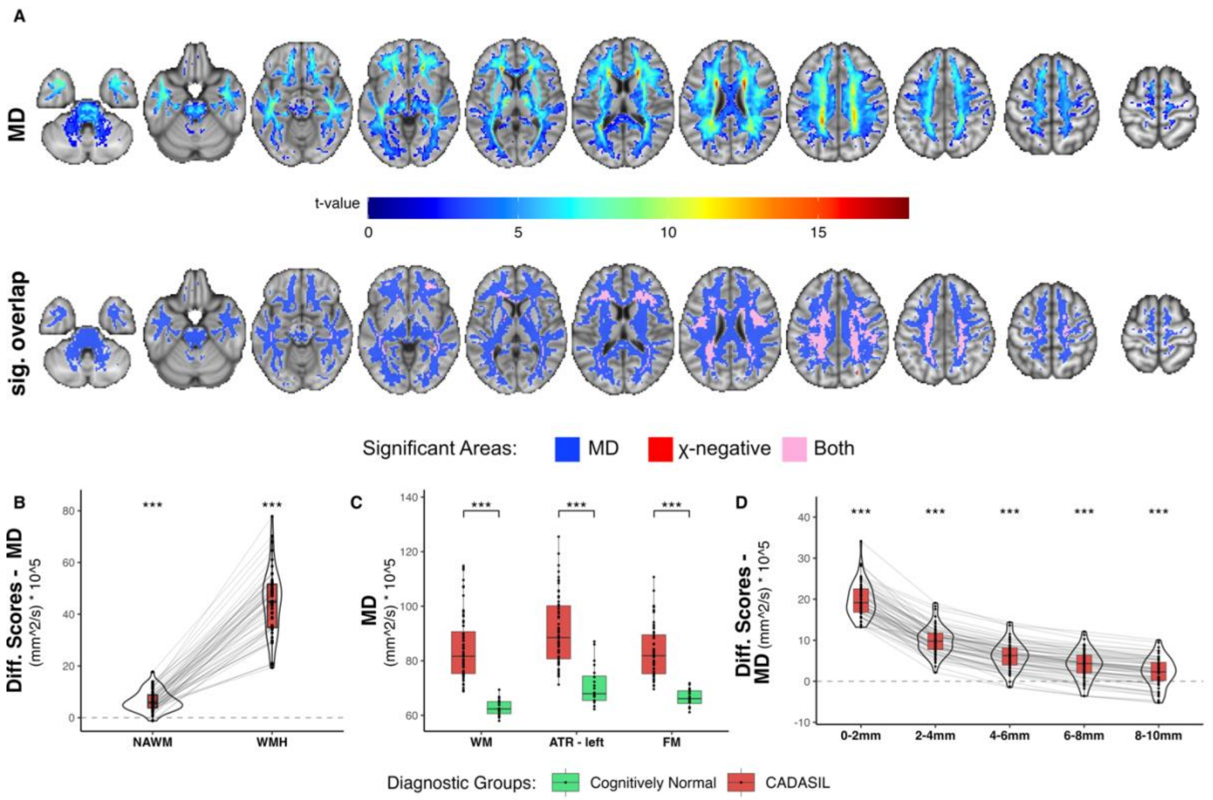

Figure E1. (A) Significant voxel-wise difference between CADASIL patients and CN of MD (CADASIL > CN) in the white matter mapped onto axial (FDR corrected $\alpha = 0.05$, voxel extent threshold of ten). Testing for the opposite contrast did not yield any significant cluster. Left side is left hemisphere. The second row visualizes the areas where either MD or χ-negative or both are significantly changed. (B) Violin blots with inserted box plots for MD difference-scores extracted from NAWM and WMH areas. Each line represents the values for a CADASIL patient. (C) Regular mean scores extracted from the global white matter, ATR-left and FM. (D) Differences scores in MD values as a function of distance from WMH areas in the CADASIL group. Difference-scores have been calculated in CADASIL from each subject specific WMH/NAWM mask after they were corrected for age, sex, education, and χ-positive. Regular scores for the white matter and the two tracts are not given as differences scores, because these areas don't vary between individuals. For NAWM, WMH, and the WMH penumbra, one-sample Welch *t*-tests ($m = 0$) were conducted and *p*-values plotted as stars (* $p < .05$, ** $p < .01$, *** $p < .001$). For the comparison of the white matter and tract scores, a regular linear model was used, with age, sex, education, and χ-positive added as covariates.

Figure E2 Voxel-wise and ROI level descriptive results for free water showing group difference in CADASIL and cognitively normal controls.

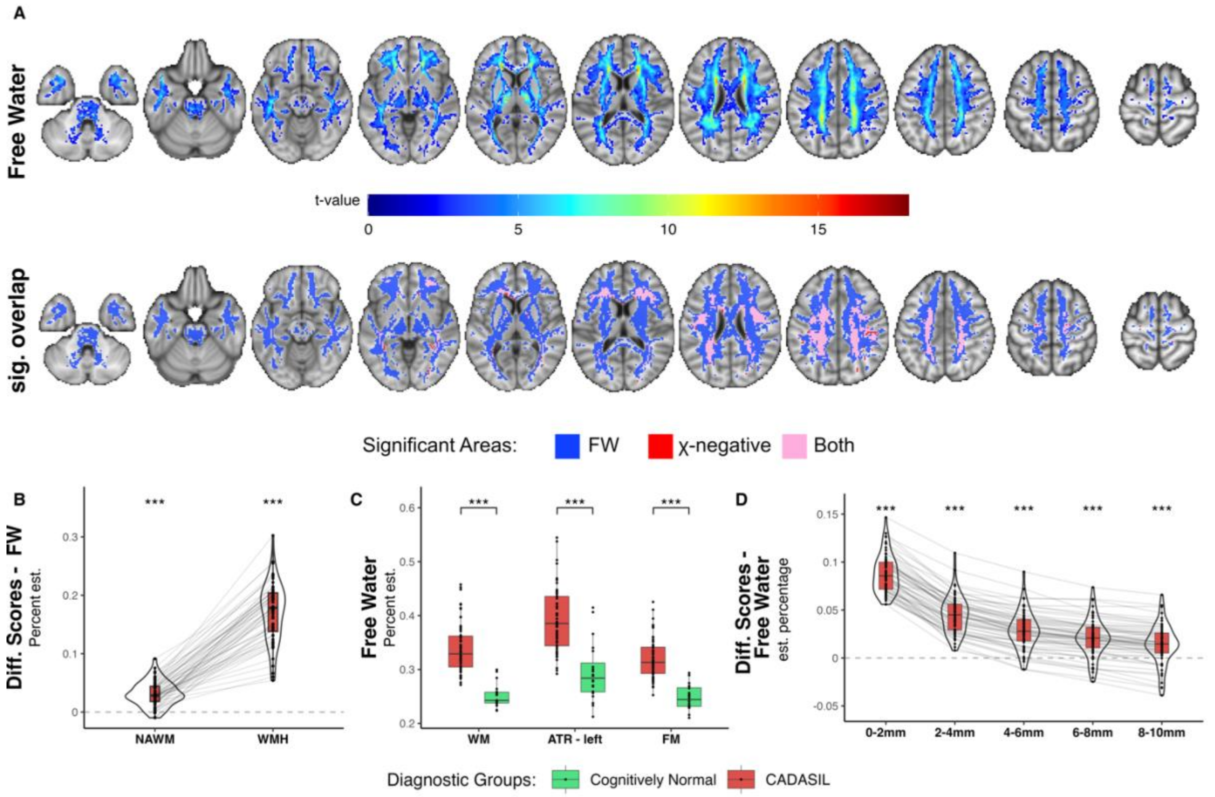

Figure E2 (A) Significant voxel-wise difference between CADASIL patients and CN of FW (CADASIL > CN) in the white matter mapped onto axial (FDR corrected $\alpha = 0.05$, voxel extent threshold of ten). Testing for the opposite contrast did not yield any significant cluster. Left side is left hemisphere. The second row visualizes the areas where either FW or χ-negative or both are significantly changed. (B) Violin blots with inserted box plots for FW difference-scores extracted from NAWM and WMH areas. Each line represents the values for a CADASIL patient. (C) Regular mean scores extracted from the global white matter, ATR-left and FM. (D) Differences scores in FW values as a function of distance from WMH areas in the CADASIL group. Difference-scores have been calculated in CADASIL from each subject specific WMH/NAWM mask after they were corrected for age, sex, education, and χ-positive. Regular scores for the white matter and the two tracts are not given as differences scores, because these areas don't vary between individuals. For NAWM, WMH, and the WMH penumbra, one-sample Welch *t*-tests ($m = 0$) were conducted and *p*-values plotted as stars (* $p < .05$, ** $p < .01$, *** $p < .001$). For the comparison of the white matter and tract scores, a regular linear model was used, with age, sex, education, and χ-positive added as covariates.

Figure E3 Voxel-wise and ROI level descriptive results for radial diffusivity showing group difference in CADASIL and cognitively normal controls.

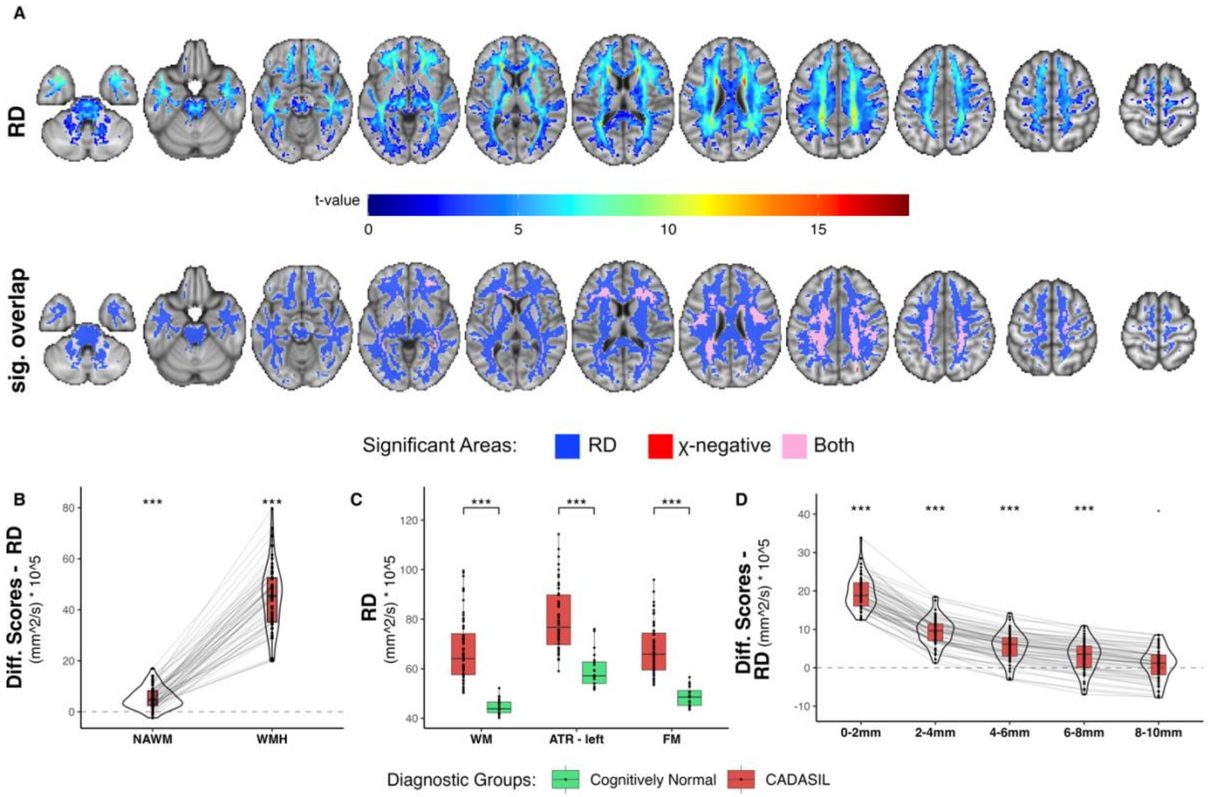

Figure E3 (A) Significant voxel-wise difference between CADASIL patients and CN of RD (CADASIL > CN) in the white matter mapped onto axial (FDR corrected $\alpha = 0.05$, voxel extent threshold of ten). Testing for the opposite contrast did not yield any significant cluster. Left side is left hemisphere. The second row visualizes the areas where either RD or χ-negative or both are significantly changed. (B) Violin blots with inserted box plots for RD difference-scores extracted from NAWM and WMH areas. Each line represents the values for a CADASIL patient. (C) Regular mean scores extracted from the global white matter, ATR-left and FM. (D) Differences scores in RD values as a function of distance from WMH areas in the CADASIL group. Difference-scores have been calculated in CADASIL from each subject specific WMH/NAWM mask after they were corrected for age, sex, education, and χ-positive. Regular scores for the white matter and the two tracts are not given as differences scores, because these areas don't vary between individuals. For NAWM, WMH, and the WMH penumbra, one-sample Welch *t*-tests ($m = 0$) were conducted and *p*-values plotted as stars (. $p < 0.1$, * $p < .05$, ** $p < .01$, *** $p < .001$). For the comparison of the white matter and tract scores, a regular linear model was used, with age, sex, education, and χ-positive added as covariates.

Figure E4 Voxel-wise and ROI level descriptive results for χ-positive showing group difference in CADASIL and cognitively normal controls.

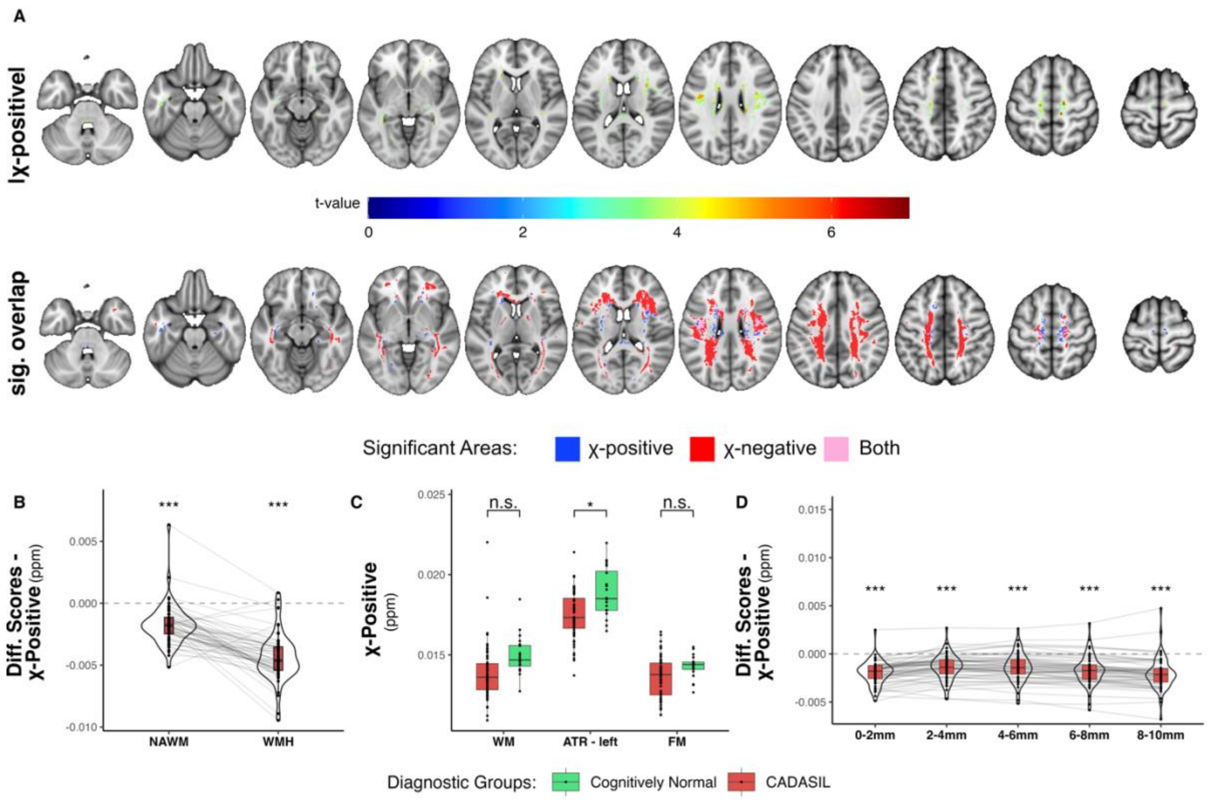

Figure E4 (A) Significant voxel-wise difference between CADASIL patients and CN of χ-positive (CADASIL < CN) in the white matter mapped onto axial (FDR corrected α = 0.05, voxel extent threshold of ten). Testing for the opposite contrast did not yield any significant cluster. Left side is left hemisphere. The second row visualizes the areas where either χ-positive or χ-negative or both are significantly changed. (B) Violin blots with inserted box plots for χ-positive difference-scores extracted from NAWM and WMH areas. Each line represents the values for a CADASIL patient. (C) Regular mean scores extracted from the global white matter, ATR-left and FM. (D) Differences scores in χ-positive values as a function of distance from WMH areas in the CADASIL group. Difference-scores have been calculated in CADASIL from each subject specific WMH/NAWM mask after they were corrected for age, sex, education, and FW. Regular scores for the white matter and the two tracts are not given as differences scores, because these areas don't vary between individuals. For NAWM, WMH, and the WMH penumbra, one-sample Welch *t*-tests ($m = 0$) were conducted and *p*-values plotted as stars (* $p < .05$, ** $p < .01$, *** $p < .001$). For the comparison of the white matter and tract scores, a regular linear model was used, with age, sex, education, and FW added as covariates.

Figure E5 Voxel-wise and ROI level descriptive results for fractional anisotropy showing group difference in CADASIL and cognitively normal controls.

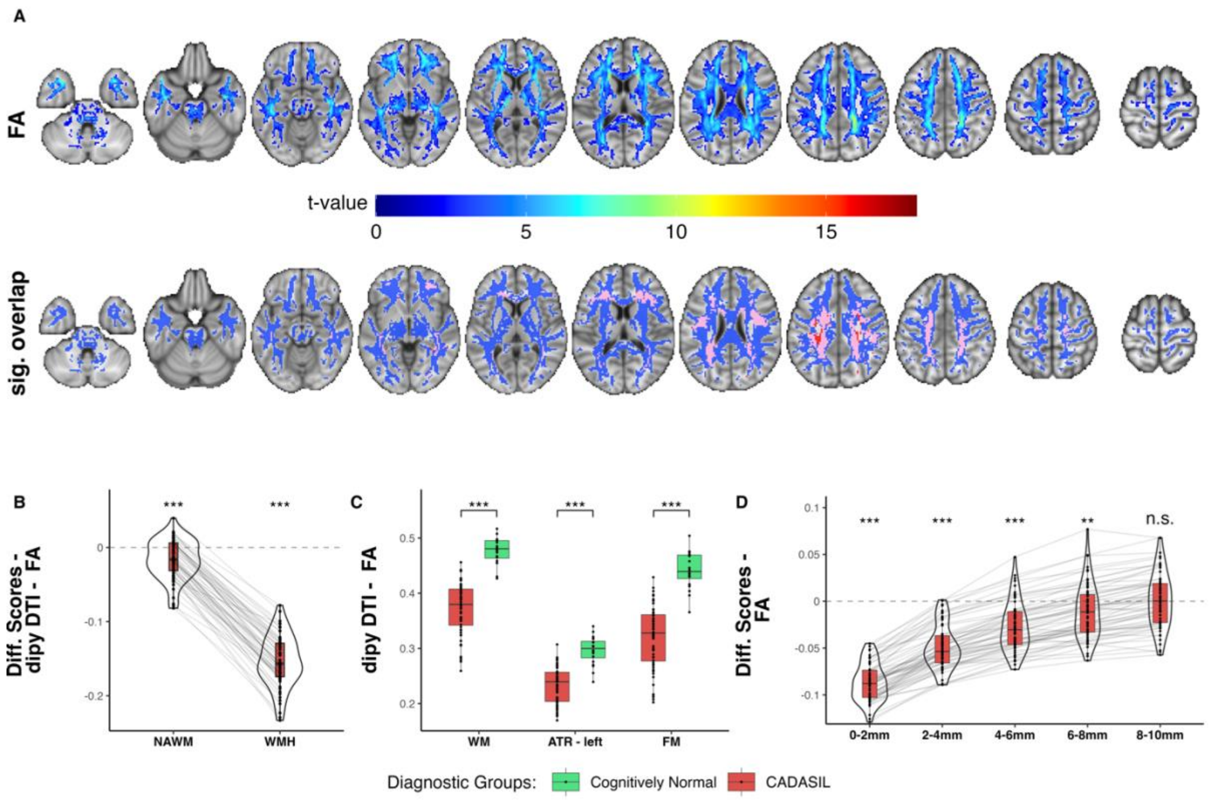

Figure E5 (A) Significant voxel-wise difference between CADASIL patients and CN of fractional anisotropy (CADASIL < CN) in the white matter mapped onto axial (FDR corrected $\alpha = 0.05$, voxel extent threshold of ten). Testing for the opposite contrast did not yield any significant cluster. Left side is left hemisphere. The second row visualizes the areas where either fractional anisotropy or χ-negative or both are significantly changed. (B) Violin blots with inserted box plots for fractional anisotropy difference-scores extracted from NAWM and WMH areas. Each line represents the values for a CADASIL patient. (C) Regular mean scores extracted from the global white matter, ATR-left and FM. (D) Differences scores in fractional anisotropy values as a function of distance from WMH areas in the CADASIL group. Difference-scores have been calculated in CADASIL from each subject specific WMH/NAWM mask after they were corrected for age, sex, education, and FW. Regular scores for the white matter and the two tracts are not given as differences scores, because these areas don't vary between individuals. For NAWM, WMH, and the WMH penumbra, one-sample Welch *t*-tests ($m = 0$) were conducted and *p*-values plotted as stars (* $p < .05$, ** $p < .01$, *** $p < .001$). For the comparison of the white matter and tract scores, a regular linear model was used, with age, sex, education, and FW added as covariates.

Table E2 Welch one-sample t-test results for the penumbra effects.

Measurement	WMH Distance	*t*	*df*	*p*	*d*	95% CI
Diff. Score MD	0-2mm	36.11	59	< .001***	4.66	[3.78, 5.52]
	2-4mm	22.22	59	< .001***	2.87	[2.29, 3.44]
	4-6mm	13.76	59	< .001***	1.78	[1.37, 2.18]
	6-8mm	8.63	59	< .001***	1.11	[0.79, 1.43]
	8-10mm	4.80	59	< .001***	0.62	[0.34, 0.89]
Diff. Score FW	0-2mm	33.28	59	< .001***	4.30	[3.48, 5.09]
	2-4mm	17.59	59	< .001***	2.27	[1.79, 2.75]
	4-6mm	11.19	59	< .001***	1.44	[1.08, 1.80]
	6-8mm	8.07	59	< .001***	1.04	[0.72, 1.35]
	8-10mm	5.22	59	< .001***	0.67	[0.39, 0.95]
Diff. Score FA	0-2mm	-32.64	59	< .001***	-4.21	[-5.00, -3.41]
	2-4mm	-14.74	59	< .001***	-1.90	[-2.33, -1.47]
	4-6mm	-6.96	59	< .001***	-0.90	[-1.20, -0.60]
	6-8mm	-2.14	59	.037*	-0.28	[-0.53, -0.02]
	8-10mm	1.28	59	.204	0.17	[-0.09, 0.42]
Diff. Score RD	0-2mm	34.60	59	< .001***	4.47	[3.62, 5.29]
	2-4mm	19.79	59	< .001***	2.55	[2.03, 3.08]
	4-6mm	11.15	59	< .001***	1.44	[1.07, 1.80]
	6-8mm	5.75	59	< .001***	0.74	[0.45, 1.03]
	8-10mm	1.72	59	.090	0.22	[-0.03, 0.48]
Diff. Score χ-positive	0-2mm	-12.14	59	< .001***	-1.57	[-1.94, -1.18]
	2-4mm	-7.82	59	< .001***	-1.01	[-1.32, -0.70]
	4-6mm	-7.43	59	< .001***	-0.96	[-1.26, -0.65]
	6-8mm	-8.69	59	< .001***	-1.12	[-1.44, -0.79]
	8-10mm	-9.36	59	< .001***	-1.21	[-1.54, -0.87]

Note. Statistical results from one-sample Welch *t*-tests ($\mu = 0$) for MD, FW, RD, and χ-positive in the penumbra of WMH. Subject specific scores were corrected for age, sex, education, and χ-positive (except when testing for χ-positive) before the calculation of the difference scores. χ-Positive scores were furthermore controlled for FW. Only NAWM without lacunar lesions were assessed. * p < .05, ** p < .01, *** p < .001

Figure E6 Association between χ-negative scores in white matter regions and various cognitive measures.

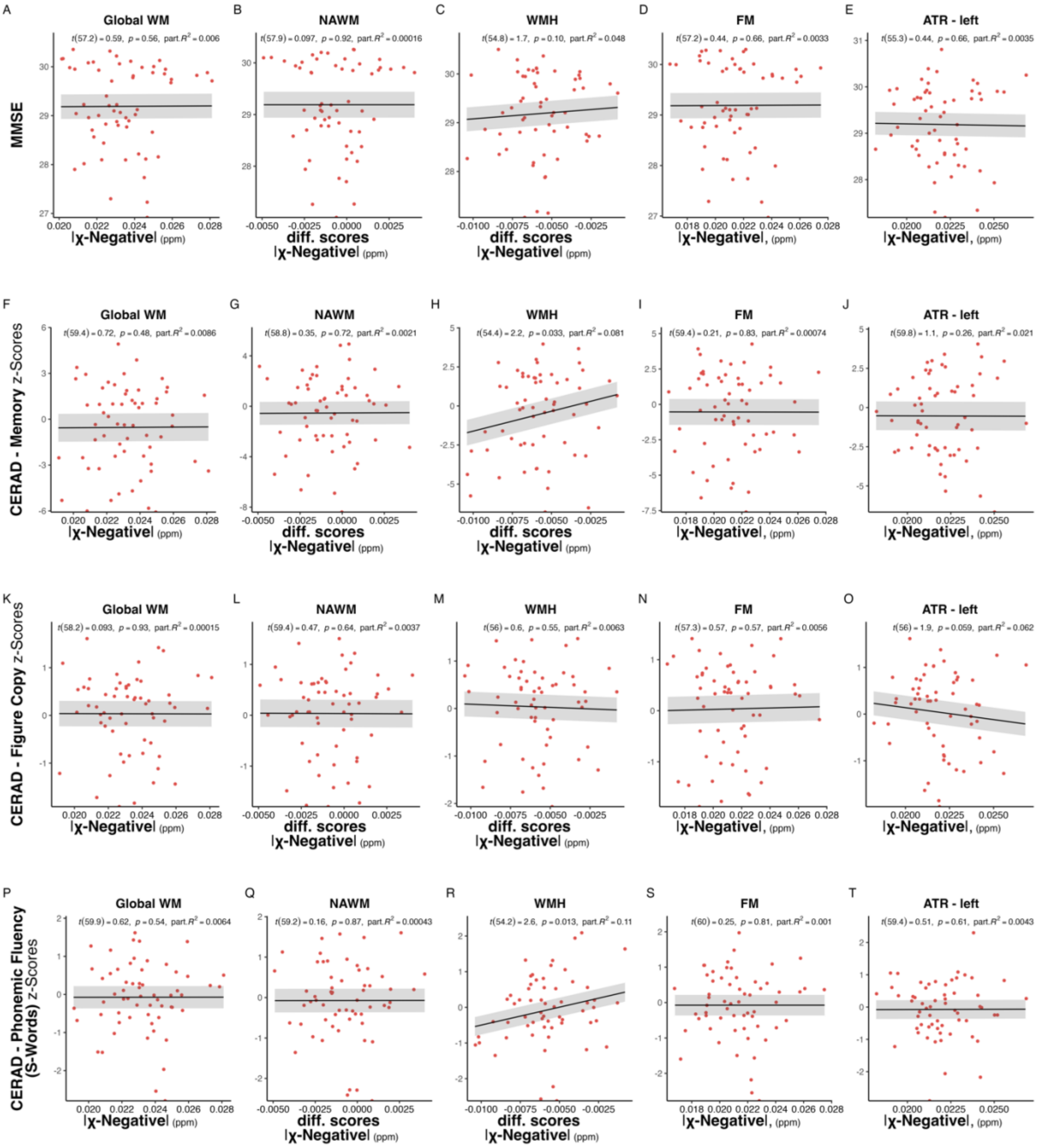

Figure E6. Residual regression plots from ridge regression models for MMSE (A – E), CERAD memory compound score (F – J), CERAD Figure Copy (K – O), CERAD S-Words (P – T), as a function of χ-negative scores in patients with CADASIL. CERAD memory compound scores are average scores of word list total, word list recall, word list recognition and figure recall. Regression line and 95% confidence level (shaded area) are shown.

Residual regression plots may indicate individuals at a higher than possible level as they are corrected for covariates.

Table E3 Statistics for predicting MMSE split by WM ROIs

WM region/Predictors	Est. (Scaled)	*SE* (Scaled)	*t*-value	*df*	*p*-value	partial R^2
**Global WM**						
|χ-negative|	0.03	0.05	0.59	57.2	.560	0.01
MD	-0.01	0.04	0.31	57.2	.761	0.00
FW	-0.01	0.04	0.20	57.2	.842	0.00
χ-positive	-0.02	0.05	0.40	57.2	.690	0.00
WMH Volume	-0.00	0.04	0.03	57.2	.978	0.00
Age (Years)	-0.04	0.05	0.77	57.2	.444	0.01
Sex (male)	-0.06	0.05	1.22	57.2	.228	0.03
Education (Years)	-0.01	0.05	0.21	57.2	.837	0.00
**NAWM**						
|χ-negative| NAWM Diff. Score	0.00	0.01	0.10	57.9	.923	0.00
MD NAWM Diff. Score	0.00	0.01	0.10	57.9	.918	0.00
FW NAWM Diff. Score	0.00	0.01	0.18	57.9	.857	0.00
χ-positive NAWM Diff. Score	0.00	0.01	0.18	57.9	.856	0.00
WMH Volume	-0.00	0.01	0.08	57.9	.939	0.00
Age (Years)	-0.01	0.01	0.78	57.9	.439	0.01
Sex (male)	-0.01	0.01	1.24	57.9	.221	0.03
Education (Years)	-0.00	0.01	0.21	57.9	.837	0.00
**WMH**						
|χ-negative| Diff. Score	0.39	0.23	1.67	54.8	.101	0.05
MD Diff. Score	-0.26	0.17	1.49	54.8	.143	0.04
FW Diff. Score	-0.24	0.18	1.36	54.8	.178	0.03
χ-positive Diff. Score	-0.07	0.22	0.33	54.8	.746	0.00
WMH Volume	0.07	0.20	0.35	54.8	.729	0.00
Age (Years)	-0.14	0.24	0.61	54.8	.542	0.01
Sex (male)	-0.27	0.25	1.08	54.8	.284	0.02
Education (Years)	-0.06	0.25	0.23	54.8	.819	0.00
**FM**						
|χ-negative|	0.02	0.05	0.44	57.2	.664	0.00
MD	-0.01	0.05	0.29	57.2	.771	0.00
FW	-0.01	0.05	0.19	57.2	.851	0.00
χ-positive	0.01	0.05	0.26	57.2	.795	0.00
WMH Volume	-0.00	0.05	0.03	57.2	.977	0.00
Age (Years)	-0.04	0.05	0.77	57.2	.442	0.01
Sex (male)	-0.06	0.05	1.23	57.2	.223	0.03
Education (Years)	-0.01	0.05	0.21	57.2	.836	0.00
**ATR-left**						
|χ-negative|	-0.09	0.20	0.44	55.3	.663	0.00
MD	-0.14	0.15	0.98	55.3	.332	0.02
FW	-0.20	0.15	1.35	55.3	.183	0.03
χ-positive	0.30	0.19	1.53	55.3	.132	0.04
WMH Volume	0.04	0.16	0.25	55.3	.804	0.00
Age (Years)	-0.10	0.18	0.54	55.3	.592	0.01
Sex (male)	-0.24	0.19	1.24	55.3	.220	0.03
Education (Years)	-0.06	0.19	0.29	55.3	.776	0.00

Note. Statistical results for MMSE. Negative values indicate worse test performance. WMH and NAWM models represents difference scores from within individual WMH and NAWM areas. Global WM, FM, and left ATR represents scores from the respective tracts, regardless of WMH or NAWM. WMH volume was standardized by the total intracranial volume. * $p < .05$, ** $p < .01$, *** $p < .001$

Table E4 Statistics for predicting CERAD memory compound score split by WM ROIs

WM region/Predictors	Est. (Scaled)	*SE* (Scaled)	*t*-value	*df*	*p*-value	partial R^2
**Global WM**						
|χ-negative|	0.10	0.14	0.72	59.4	.475	0.01
MD	-0.06	0.13	0.44	59.4	.660	0.00
FW	0.05	0.13	0.34	59.4	.733	0.00
χ-positive	-0.23	0.14	1.60	59.4	.116	0.04
WMH Volume	0.13	0.13	0.96	59.4	.343	0.02
Age (Years)	0.15	0.14	1.09	59.4	.282	0.02
Sex (male)	0.13	0.14	0.92	59.4	.360	0.01
Education (Years)	-0.14	0.14	0.97	59.4	.336	0.02
**NAWM**						
|χ-negative| NAWM Diff. Score	0.10	0.27	0.35	58.8	.725	0.00
MD NAWM Diff. Score	-0.26	0.25	1.02	58.8	.313	0.02
FW NAWM Diff. Score	0.19	0.26	0.73	58.8	.467	0.01
χ-positive NAWM Diff. Score	-0.15	0.27	0.56	58.8	.579	0.01
WMH Volume	0.24	0.26	0.92	58.8	.364	0.01
Age (Years)	0.29	0.27	1.07	58.8	.288	0.02
Sex (male)	0.25	0.27	0.91	58.8	.366	0.01
Education (Years)	-0.27	0.27	0.98	58.8	.331	0.02
**WMH**						
|χ-negative| Diff. Score	4.14	1.89	2.19	54.4	.033*	0.08
MD Diff. Score	-3.74	1.34	2.80	54.4	.007**	0.13
FW Diff. Score	0.06	1.36	0.04	54.4	.966	0.00
χ-positive Diff. Score	-2.87	1.92	1.49	54.4	.141	0.04
WMH Volume	2.11	1.79	1.18	54.4	.243	0.03
Age (Years)	2.46	1.99	1.24	54.4	.220	0.03
Sex (male)	3.01	1.98	1.52	54.4	.134	0.04
Education (Years)	-2.02	1.98	1.02	54.4	.312	0.02
**FM**						
|χ-negative|	-0.03	0.13	0.21	59.4	.834	0.00
MD	-0.09	0.12	0.75	59.4	.457	0.01
FW	-0.02	0.12	0.19	59.4	.851	0.00
χ-positive	-0.03	0.13	0.21	59.4	.834	0.00
WMH Volume	0.12	0.12	0.96	59.4	.342	0.02
Age (Years)	0.14	0.13	1.09	59.4	.281	0.02
Sex (male)	0.12	0.13	0.90	59.4	.373	0.01
Education (Years)	-0.13	0.13	0.98	59.4	.333	0.02
**ATR-left**						
|χ-negative|	-0.04	0.04	1.14	59.8	.258	0.02
MD	-0.05	0.04	1.37	59.8	.175	0.03
FW	-0.04	0.04	1.12	59.8	.267	0.02
χ-positive	-0.04	0.04	1.08	59.8	.284	0.02
WMH Volume	0.03	0.04	0.94	59.8	.353	0.01
Age (Years)	0.04	0.04	1.10	59.8	.278	0.02
Sex (male)	0.03	0.04	0.90	59.8	.372	0.01
Education (Years)	-0.04	0.04	0.97	59.8	.334	0.02

Note. Statistical results for CERAD memory compound score, comprised of word list total, word list recall, word list recognition and figure recall. Negative values indicate worse test performance. WMH and NAWM models represents difference scores from within individual WMH and NAWM areas. Global WM, FM, and left ATR represents scores from the respective tracts, regardless of WMH or NAWM. WMH volume was standardized by the total intracranial volume. * $p < .05$, ** $p < .01$, *** $p < .001$

Table E5 Statistics for predicting CERAD Figure Copy split by WM ROIs

WM region/Predictors	Est. (Scaled)	*SE* (Scaled)	*t*-value	*df*	*p*-value	partial R^2
**Global WM**						
|χ-negative|	-0.01	0.13	0.09	58.2	.926	0.00
MD	-0.15	0.11	1.41	58.2	.164	0.03
FW	-0.07	0.11	0.66	58.2	.510	0.01
χ-positive	-0.34	0.13	2.51	58.2	.015*	0.10
WMH Volume	-0.03	0.11	0.28	58.2	.781	0.00
Age (Years)	-0.10	0.13	0.81	58.2	.422	0.01
Sex (male)	0.12	0.13	0.86	58.2	.392	0.01
Education (Years)	-0.01	0.14	0.10	58.2	.920	0.00
**NAWM**						
|χ-negative| NAWM Diff. Score	-0.02	0.04	0.47	59.4	.642	0.00
MD NAWM Diff. Score	-0.02	0.04	0.51	59.4	.610	0.00
FW NAWM Diff. Score	0.04	0.04	1.08	59.4	.285	0.02
χ-positive NAWM Diff. Score	-0.10	0.04	2.56	59.4	.013*	0.10
WMH Volume	-0.02	0.04	0.39	59.4	.698	0.00
Age (Years)	-0.03	0.04	0.86	59.4	.393	0.01
Sex (male)	0.03	0.04	0.74	59.4	.460	0.01
Education (Years)	-0.00	0.04	0.08	59.4	.940	0.00
**WMH**						
|χ-negative| Diff. Score	-0.21	0.36	0.60	56	.553	0.01
MD Diff. Score	-0.57	0.25	2.26	56	.028*	0.08
FW Diff. Score	-0.35	0.26	1.37	56	.177	0.03
χ-positive Diff. Score	-0.50	0.35	1.46	56	.150	0.04
WMH Volume	0.21	0.31	0.68	56	.502	0.01
Age (Years)	-0.21	0.37	0.56	56	.574	0.01
Sex (male)	0.41	0.38	1.08	56	.286	0.02
Education (Years)	-0.08	0.38	0.20	56	.841	0.00
**FM**						
|χ-negative|	0.12	0.21	0.57	57.3	.573	0.01
MD	-0.26	0.16	1.60	57.3	.116	0.04
FW	-0.11	0.17	0.66	57.3	.509	0.01
χ-positive	-0.26	0.22	1.19	57.3	.238	0.02
WMH Volume	-0.01	0.18	0.07	57.3	.946	0.00
Age (Years)	-0.16	0.21	0.75	57.3	.458	0.01
Sex (male)	0.20	0.22	0.90	57.3	.369	0.01
Education (Years)	-0.04	0.22	0.20	57.3	.844	0.00
**ATR-left**						
|χ-negative|	-0.68	0.35	1.92	56	.059	0.06
MD	-0.64	0.24	2.66	56	.010*	0.11
FW	-0.45	0.25	1.79	56	.079	0.05
χ-positive	0.38	0.35	1.09	56	.279	0.02
WMH Volume	0.13	0.30	0.45	56	.651	0.00
Age (Years)	-0.07	0.32	0.22	56	.825	0.00
Sex (male)	0.29	0.35	0.83	56	.410	0.01
Education (Years)	-0.10	0.35	0.28	56	.780	0.00

Note. Statistical results for CERAD Figure Copy. Negative values indicate worse test performance. WMH and NAWM models represents difference scores from within individual WMH and NAWM areas. Global WM, FM, and left ATR represents scores from the respective tracts, regardless of WMH or NAWM. WMH volume was standardized by the total intracranial volume. * $p < .05$, ** $p < .01$, *** $p < .001$

Table E6 Statistics for predicting CERAD Phonemic Fluency (S-Words) split by WM ROIs

WM region/Predictors	Est. (Scaled)	*SE* (Scaled)	*t*-value	*df*	*p*-value	partial R^2
**Global WM**						
|χ-negative|	0.01	0.01	0.62	59.9	.537	0.01
MD	-0.01	0.01	0.61	59.9	.547	0.01
FW	0.00	0.01	0.00	59.9	.996	0.00
χ-positive	-0.01	0.01	1.32	59.9	.192	0.03
WMH Volume	0.01	0.01	0.62	59.9	.537	0.01
Age (Years)	0.01	0.01	1.38	59.9	.173	0.03
Sex (male)	0.00	0.01	0.29	59.9	.774	0.00
Education (Years)	-0.01	0.01	0.67	59.9	.507	0.01
**NAWM**						
|χ-negative| NAWM Diff. Score	0.01	0.05	0.16	59.2	.874	0.00
MD NAWM Diff. Score	-0.05	0.05	1.08	59.2	.287	0.02
FW NAWM Diff. Score	0.04	0.05	0.69	59.2	.494	0.01
χ-positive NAWM Diff. Score	-0.02	0.05	0.42	59.2	.680	0.00
WMH Volume	0.03	0.05	0.61	59.2	.544	0.01
Age (Years)	0.07	0.05	1.39	59.2	.170	0.03
Sex (male)	0.02	0.05	0.28	59.2	.777	0.00
Education (Years)	-0.04	0.05	0.67	59.2	.508	0.01
**WMH**						
|χ-negative| Diff. Score	1.64	0.64	2.56	54.2	.013*	0.11
MD Diff. Score	-0.50	0.46	1.10	54.2	.278	0.02
FW Diff. Score	0.15	0.46	0.33	54.2	.746	0.00
χ-positive Diff. Score	-1.02	0.65	1.56	54.2	.124	0.04
WMH Volume	-0.02	0.61	0.03	54.2	.979	0.00
Age (Years)	1.10	0.67	1.65	54.2	.106	0.05
Sex (male)	0.45	0.67	0.67	54.2	.507	0.01
Education (Years)	-0.34	0.66	0.51	54.2	.612	0.00
**FM**						
|χ-negative|	0.00	0.00	0.25	60	.805	0.00
MD	-0.00	0.00	0.94	60	.349	0.01
FW	-0.00	0.00	0.45	60	.655	0.00
χ-positive	0.00	0.00	0.27	60	.788	0.00
WMH Volume	0.00	0.00	0.61	60	.542	0.01
Age (Years)	0.00	0.00	1.37	60	.175	0.03
Sex (male)	0.00	0.00	0.28	60	.777	0.00
Education (Years)	-0.00	0.00	0.67	60	.506	0.01
**ATR-left**						
|χ-negative|	0.02	0.04	0.51	59.4	.615	0.00
MD	-0.06	0.04	1.55	59.4	.126	0.04
FW	-0.05	0.04	1.39	59.4	.169	0.03
χ-positive	-0.06	0.04	1.45	59.4	.151	0.03
WMH Volume	0.03	0.04	0.70	59.4	.485	0.01
Age (Years)	0.05	0.04	1.45	59.4	.153	0.03
Sex (male)	0.01	0.04	0.31	59.4	.755	0.00
Education (Years)	-0.03	0.04	0.67	59.4	.503	0.01

Note. Statistical results for CERAD Phonemic Fluency (S-Words). Negative values indicate worse test performance. WMH and NAWM models represents difference scores from within individual WMH and NAWM areas. Global WM, FM, and left ATR represents scores from the respective tracts, regardless of WMH or NAWM. WMH volume was standardized by the total intracranial volume. * $p < .05$, ** $p < .01$, *** $p < .001$

Figure E7. Native space differences scores in susceptibility and DWI scores as a function of distance from WMH areas in the CADASIL group.

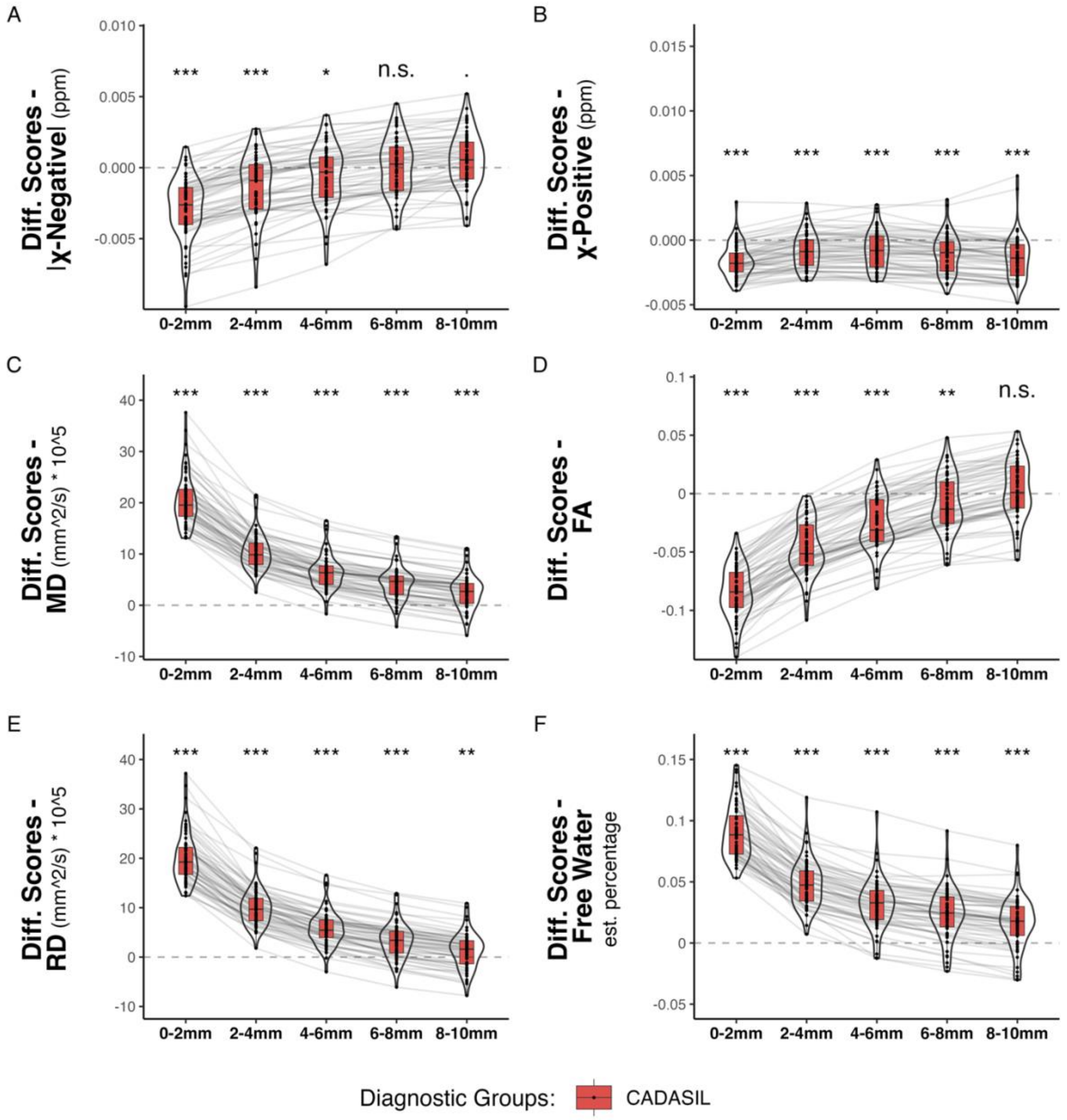

Figure E7. Difference-scores have been calculated in CADASIL from each subject specific penumbra mask in native space and subtracted by HC scores in MNI space. Therefore, no warping of the CADASIL images is required and the effect of smoothing by resampling is ruled out. One-sample Welch *t*-tests ($m = 0$) were conducted and *p*-values plotted as stars (* $p < .05$, ** $p < .01$, *** $p < .001$).

Table E7 Welch one-sample t-test results for the penumbra effects in native space.

Metric	Dependent Variable	*t*	*df*	*p*	*d*	95% CI
Diff. Score |χ-negative|	0-2mm	-10.03	59	< .001***	-1.30	[-1.64, -0.95]
	2-4mm	-4.74	59	< .001***	-0.61	[-0.89, -0.33]
	4-6mm	-2.19	59	.032*	-0.28	[-0.54, -0.02]
	6-8mm	0.11	59	.910	0.01	[-0.24, 0.27]
	8-10mm	1.87	59	.067	0.24	[-0.02, 0.50]
Diff. Score χ-positive	0-2mm	-9.67	59	< .001***	-1.25	[-1.58, -0.91]
	2-4mm	-4.69	59	< .001***	-0.61	[-0.88, -0.33]
	4-6mm	-4.25	59	< .001***	-0.55	[-0.82, -0.27]
	6-8mm	-5.63	59	< .001***	-0.73	[-1.01, -0.44]
	8-10mm	-6.26	59	< .001***	-0.81	[-1.10, -0.51]
Diff. Score MD	0-2mm	31.10	59	< .001***	4.01	[3.25, 4.78]
	2-4mm	22.74	59	< .001***	2.94	[2.35, 3.52]
	4-6mm	15.00	59	< .001***	1.94	[1.50, 2.36]
	6-8mm	9.83	59	< .001***	1.27	[0.93, 1.61]
	8-10mm	5.85	59	< .001***	0.76	[0.47, 1.04]
Diff. Score RD	0-2mm	29.67	59	< .001***	3.83	[3.09, 4.56]
	2-4mm	20.51	59	< .001***	2.65	[2.11, 3.18]
	4-6mm	12.66	59	< .001***	1.63	[1.24, 2.02]
	6-8mm	6.99	59	< .001***	0.90	[0.60, 1.20]
	8-10mm	2.77	59	.008**	0.36	[0.09, 0.62]
Diff. Score FA	0-2mm	-29.81	59	< .001***	-3.85	[-4.58, -3.11]
	2-4mm	-15.86	59	< .001***	-2.05	[-2.49, -1.60]
	4-6mm	-8.43	59	< .001***	-1.09	[-1.41, -0.77]
	6-8mm	-2.87	59	.006**	-0.37	[-0.63, -0.11]
	8-10mm	0.82	59	.416	0.11	[-0.15, 0.36]
Diff. Score FW	0-2mm	32.28	59	< .001***	4.17	[3.37, 4.94]
	2-4mm	19.34	59	< .001***	2.50	[1.98, 3.01]
	4-6mm	12.24	59	< .001***	1.58	[1.20, 1.96]
	6-8mm	9.06	59	< .001***	1.17	[0.84, 1.50]
	8-10mm	6.22	59	< .001***	0.80	[0.51, 1.09]

Note. Statistical results from one-sample Welch *t*-tests ($\mu = 0$) for MD, FA, FW, RD, χ-negative and χ-positive in the penumbra of WMH. Only NAWM without lacunar lesions were assessed. * p < .05, ** p < .01, *** p < .001
